# Efficacy of Q-Switched and picosecond lasers for acquired bilateral nevus of Ota-like macules: a systematic review and exploratory dose-response meta-analysis

**DOI:** 10.1007/s10103-026-04982-6

**Published:** 2026-08-06

**Authors:** Feng Li, Yufeng Wang, Zhaoxia Shen

**Affiliations:** 1 Changzhou Dean Hospital, Changzhou, China; 2 Lianyungang Hospital of Jiangsu Province Hospital of Chinese Medicine, Lianyungang, China

**Keywords:** ABNOM, Picosecond laser, Q-switched laser, Dose-response meta-analysis, Systematic review, Laser safety

## Abstract

Acquired bilateral nevus of Ota-like macules (ABNOM) is a common dermal melanocytosis predominantly affecting East Asian women. Q-switched (QS) and picosecond (PS) lasers are widely used, but their comparative efficacy and dose-response relationships have not been quantified. We conducted the first systematic review and dose-response meta-analysis to compare PS versus QS laser efficacy for isolated ABNOM. Eleven databases were searched from inception to May 27, 2026. Forty studies (50 treatment arms) of predominantly single-arm case series were included. The primary analysis used generalized linear mixed models (GLMM) for ≥ 75% clearance, complemented by dose-response meta-regression with cluster-robust standard errors. Pooled ≥ 75% clearance was 47.8% (95% CI 13.4–84.4) for PS and 53.1% (95% CI 30.3–74.6) for QS lasers (*P* = 0.95). Each additional treatment session was associated with a 3-fold increased odds of clearance (OR 3.2; 95% CI 2.4–4.3; *P* < 0.001). As an exploratory analysis, the dose-response relationship was monotonic across sessions 1–7. The PS estimate was fragile, ranging from 47.8% to 88.7% across sensitivity analyses. GRADE evidence certainty was very low. Treatment session number, rather than laser type, appeared more strongly associated with clearance. However, this dose-response finding remains hypothesis-generating due to very low certainty of evidence and requires validation in randomized trials. The absence of a statistically significant difference between laser types does not establish equivalence; PS efficacy estimates were highly fragile and based on sparse data. Wavelength may be an independent determinant of clearance and warrants further comparative study.

**Supplementary Information:**

The online version contains supplementary material available at 10.1007/s10103-026-04982-6.

## Introduction

Acquired bilateral nevus of Ota-like macules (ABNOM, also termed Hori nevus) is a common dermal melanocytosis affecting up to 2.5% of middle-aged East Asian women [1, 2]. Unlike nevus of Ota—which involves deeper dermal melanocytes and may involve the ocular and oral mucosa—ABNOM presents as bilateral blue-brown to slate-gray macules confined to the malar, temporal, and nasal regions, with melanocytes distributed in the upper to mid-dermis [3]. ABNOM causes notable psychosocial distress among affected patients, leading to high clinical demand for safe and effective laser interventions.

Since the 1990 s, Q-switched (QS) lasers operating at nanosecond pulse durations have been the cornerstone of ABNOM management, targeting dermal melanin through selective photothermolysis [4]. The introduction of picosecond (PS) lasers in 2012 reduced pulse duration by three orders of magnitude, theoretically enabling more efficient melanosome fragmentation through photomechanical rather than photothermal disruption [5]. This technological shift generated considerable clinical enthusiasm, with numerous single-center series reporting favorable outcomes for both modalities. However, head-to-head comparisons have yielded inconsistent conclusions: some studies suggest PS superiority [6], while others find no meaningful difference after an adequate treatment course [7, 8].

A 2020 systematic review by Kaur et al. qualitatively summarized 19 ABNOM laser studies but provided no quantitative synthesis [4]. Critically, no previous work has formally modeled the relationship between cumulative treatment sessions and clearance—a question of direct clinical relevance, given that ABNOM typically requires three to six sessions spaced months apart [9].

The present study addresses this gap through two complementary analyses. First, we pool ≥ 75% clearance rates for PS and QS lasers in a conventional proportion meta-analysis. Second, and more importantly, we employ dose-response meta-regression to isolate the effect of treatment session number from that of laser type. This design tests a hypothesis with immediate clinical implications: if both laser types exhibit parallel session-response curves, then the conflicting comparative findings in the literature may simply reflect differences in treatment intensity rather than genuine platform superiority—a question that has not been previously addressed through quantitative synthesis.

## Methods

### Protocol and registration

This systematic review followed the Preferred Reporting Items for Systematic Reviews and Meta-Analyses (PRISMA) 2020 guidelines [10] and was prospectively registered with the International Prospective Register of Systematic Reviews (PROSPERO; CRD420261404917). The complete protocol (version 2.3) is provided in Appendix S1; protocol deviations are reported in Sect. 2.6 and detailed in Appendix S2.

### Search strategy

Eleven databases were searched from inception to May 27, 2026: PubMed, Embase, Cochrane CENTRAL, Web of Science, CNKI, Wanfang, CNKI/CDMD (dissertations), KoreaMed, KCI, KMBASE, and J-STAGE. ClinicalTrials.gov and ChiCTR were additionally searched to identify unpublished or ongoing trials. Full search strategies for each database are provided in Appendix S3. Scopus was not searched due to institutional access constraints; Google Scholar was precluded by regional access restrictions. The four core databases (PubMed, Embase, Web of Science, and Cochrane CENTRAL) collectively retrieved 672 records, making these omissions unlikely to have introduced meaningful bias.

Disease terms encompassed “acquired bilateral nevus of Ota-like macules,” “Hori nevus,” “Hori’s nevus,” “nevus of Hori,” “ABNOM,” “ABNOMs,” “acquired dermal melanocytosis,” “ADM,” and “facial melanocytosis.” In East Asian dermatology, ADM is effectively synonymous with ABNOM; studies retrieved solely by the ADM term were manually verified against the ABNOM case definition to exclude non-ABNOM dermal melanocytoses. Intervention terms covered Q-switched, picosecond, and nanosecond lasers, together with specific wavelengths (Nd: YAG, alexandrite, ruby, titanium sapphire). No language restrictions were imposed.

### Eligibility criteria

Studies were eligible if they met all of the following: (1) patients diagnosed with ABNOM; (2) treatment with PS or QS laser; (3) sample size *N* ≥ 5; (4) reported clearance rates with quantifiable, percentage-based thresholds (≥ 75% or ≥ 90%). Studies reporting only aggregate “overall effectiveness” without defined percentage cut-offs were excluded. Mixed-population studies (e.g., ABNOM combined with melasma) were excluded unless ABNOM-specific clearance data could be isolated.

### Study selection and data extraction

Two reviewers ([A] and [B]) independently screened titles, abstracts, and full-text articles. Prior to formal screening, 10 randomly selected articles were used for pilot calibration to unify inclusion criteria. Disagreements were resolved through consensus discussion. Inter-rater agreement at the full-text stage was fair (Cohen’s κ = 0.33), reflecting the first reviewer’s intentionally inclusive screening strategy.

Both reviewers independently extracted data using a standardized form covering study design, sample size, laser type and wavelength, treatment parameters, session schedule, cumulative clearance rates at each session, and adverse events. Clearance data were extracted only when the original grading boundary matched the target threshold exactly. Studies reporting 100% clearance in all patients were included in both the ≥ 75% and ≥ 90% pools. When a study contributed two independent treatment arms, each arm was extracted and analyzed separately.

### Risk of bias assessment

The Joanna Briggs Institute (JBI) Checklist for Case Series (10 items) was applied to all studies [11]. Each item was scored as “Yes” (1) or “No” (0). Overall risk of bias was classified as Low (≥ 7/10), Moderate (4–6/10), or High (< 4/10).

### Protocol deviations

The following deviations from the registered protocol occurred during study execution. (1) The per-session dose-response meta-regression, the standalone ≥ 90% clearance analysis, and the formal GRADE certainty assessment were added post-registration based on data availability and are reported transparently. The registered protocol had precluded continuous meta-regression for treatment sessions due to reverse causality; given the exploratory nature of this analysis and the use of cluster-robust standard errors, this restriction was relaxed. (2) Safety outcomes (PIH incidence and pigmentary changes) were registered as co-primary endpoints but could not be quantitatively synthesized because adverse event reporting across the predominantly single-arm case series was inconsistent in definition, assessment timing, and denominator—precluding valid pooling; safety data are instead summarized narratively in Sect. 4.4. (3) Of the four risk-of-bias tools specified (RoB 2, JBI, NOS, ROBINS-I), only the JBI Checklist was applied. RoB 2 is designed for RCTs [12], NOS for cohort and case-control studies, and ROBINS-I for non-randomized interventional studies; none are applicable to the predominantly case-series evidence base. (4) The registered clearance threshold (> 75%) was relaxed to ≥ 75% to align with the primary literature, where the vast majority of studies use the inclusive boundary—a pragmatically necessary decision given that physician-assessed pigment clearance is inherently imprecise at single-percentage-point resolution. (5) The registered requirement for Cohen’s κ ≥ 0.75 at the screening stage was not met (observed κ = 0.33). This reflected the first reviewer’s intentionally more inclusive screening strategy rather than unreliability; all disagreements were resolved through consensus discussion, and the impact on the final included set is negligible. (6) The PROSPERO protocol specified a three-tier evidence synthesis framework (RCT direct comparison, single-arm proportion meta-analysis with meta-regression, and safety meta-analysis); levels 2 and 3 are reported, while level 1 formal direct comparison was precluded by the small number of split-face RCTs meeting identical threshold criteria. (7) The prespecified combination-therapy subgroup was not analyzed because pharmacological treatment currently has no evidence-based role in ABNOM. No registered analyses were omitted.

### Statistical analysis

All analyses were conducted in R version 4.5.3 using the meta (version 8.3-0) and metafor (version 4.8-0) packages.

#### **Overall Comparison**

The primary analysis used GLMM with logit link and random-effects structure, robust to boundary proportions. Between-group differences were assessed via Wald test. For sensitivity, we applied PFT transformation. Pooled estimates and 95% CIs were calculated for both approaches.

#### **Dose-Response Meta-Regression**

Studies reporting cumulative clearance rates at multiple treatment sessions were analyzed with mixed-effects meta-regression: logit(clearance) ~ sessions + threshold (≥ 75% or ≥ 90%) + laser type + (1 | study). The threshold indicator was included as a covariate to prevent confounding of the session effect by outcome definition. A continuity correction of 0.5 was added to treatment arms with zero events or 100% clearance rates to enable valid logit transformation in the mixed-effects model. Only cumulative clearance data at each treatment session were included; overall pooled estimates from individual studies were excluded to avoid double-counting of participants. To address within-study serial correlation from repeated cumulative measurements, cluster-robust standard errors (cluster = study) were computed using the CR1-type estimator with small-sample adjustment as implemented in metafor::robust(). A random-slopes model (random = ~ sessions | study) was fitted as a sensitivity check. Studies reporting session data only as attainment counts without denominators were excluded.

#### Supplementary and Sensitivity Analyses

A separate proportion meta-analysis was conducted for ≥ 90% clearance. Publication bias was evaluated with Peters’ test and visualized via funnel plots; trim-and-fill analysis was also performed. Six sensitivity analyses were performed: (0) PFT transformation; (1) exclusion of moderate risk-of-bias (ROB) studies; (2) exclusion of *N* < 20; (3) exclusion of a study that pooled mixed wavelengths; (4) exclusion of studies with 0% clearance arms; and (5) leave-one-out influence analysis (Figure S3). Full model diagnostics (heterogeneity, fit statistics, and convergence status) are provided in Supplementary Table S4.

### Certainty of evidence

The Grading of Recommendations Assessment, Development and Evaluation (GRADE) framework was applied [13]. Starting from “very low” (given the predominance of single-arm case series), evidence was assessed across five domains. Upgrading was considered for large effect magnitude and dose-response gradient.

## Results

### Study selection and characteristics

**Fig. 1:**
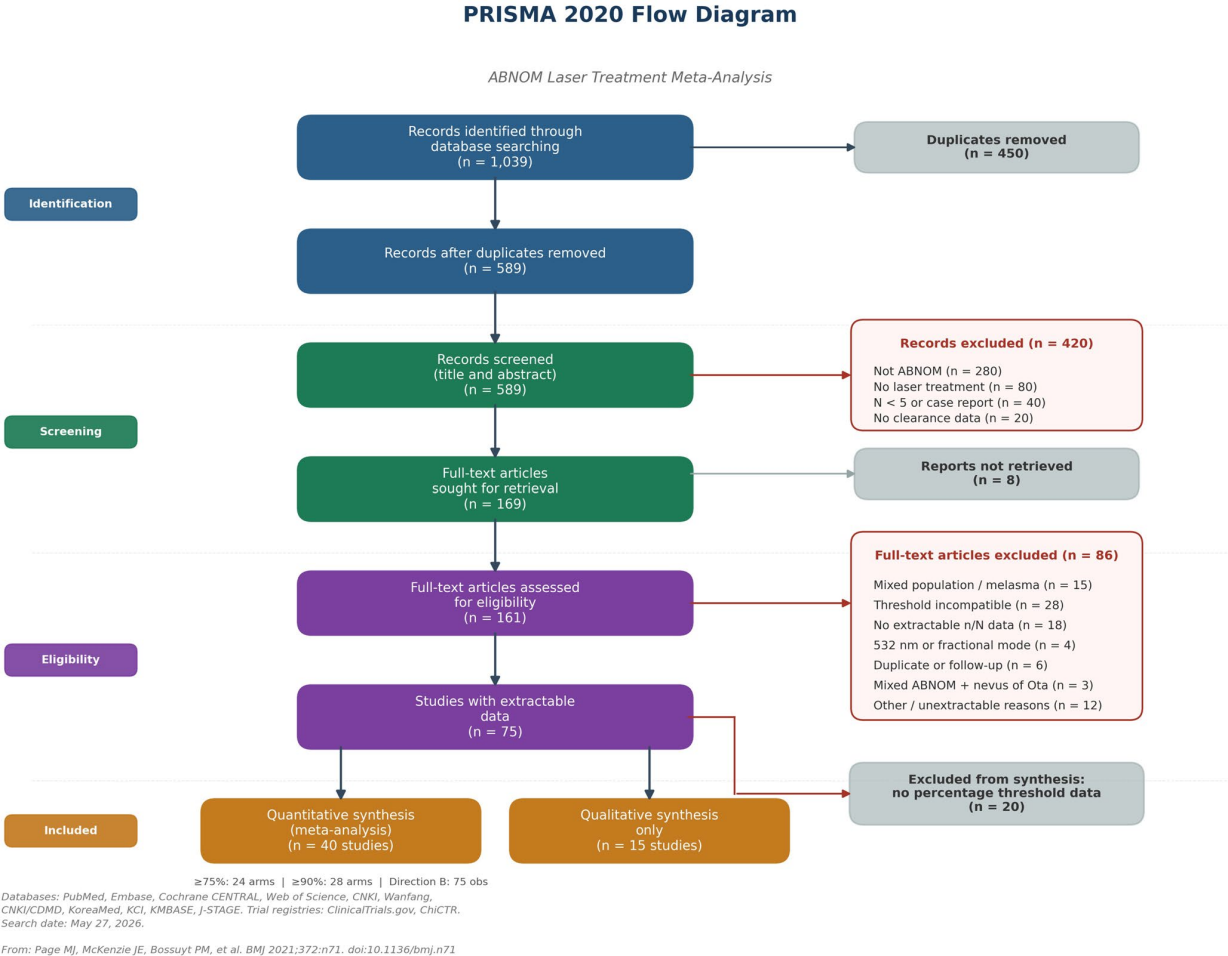
PRISMA 2020 flow diagram

Forty unique studies met all inclusion criteria (Fig. 1). An additional 15 studies satisfied eligibility criteria but reported clearance only as aggregate effectiveness summaries without extractable percentage-based thresholds; these were retained in the systematic review but excluded from quantitative synthesis. The 40 studies contributing quantitative data yielded 50 unique treatment arms (PS, 11; QS, 40, with one arm contributing to both counts). Study characteristics are summarized in Table 1. Detailed study-level characteristics are provided in Supplementary Table S5. A detailed list of 35 studies excluded at the full-text assessment and data extraction stages for specific reasons (e.g., incompatible thresholds, mixed populations) is provided in Supplementary Table S1. The complete PRISMA exclusion flow (86 studies excluded at full-text assessment) is shown in Fig. 1. The 40 studies enrolled 5,296 patients in total; 780 patients were treated in studies using PS lasers and 4,913 in studies using QS lasers (comparative studies contribute to both counts).

**Table 1:** Characteristics of included studies

Characteristic	Overall	PS	QS
Studies, n	40	10	35
Treatment arms, n	50	11	40
Patients, n	5,296†	780	4,913
Study design (RCT/Prosp/Retro), n	9/3/28	—	—
Country (China/Other), n	35/5	—	—
Sample size, median (range)	73 (5–890)	60 (5–225)	80 (5–890)
Publication years	2000–2025	2018–2025	2000–2025
Wavelengths	670–1064 nm	755 nm dominant; 670, 730, 1064 nm	1064 nm dominant; 694, 755 nm
Risk of bias (Low/Moderate), n	38/2	—	—

*PS* picosecond, *QS* Q-switched, *RCT* randomized controlled trial, *Prosp* prospective. †Patient totals in the PS and QS columns include overlapping patients from five comparative (two-arm) studies; the overall patient count excludes these duplicates. — indicates that the characteristic is reported for the overall study set only, as a laser-type-specific breakdown was not available for this variable. One treatment arm (Yu et al., 2018) contributed to both PS and QS columns; arm totals therefore do not sum

### Overall ≥ 75% clearance

**Fig. 2:**
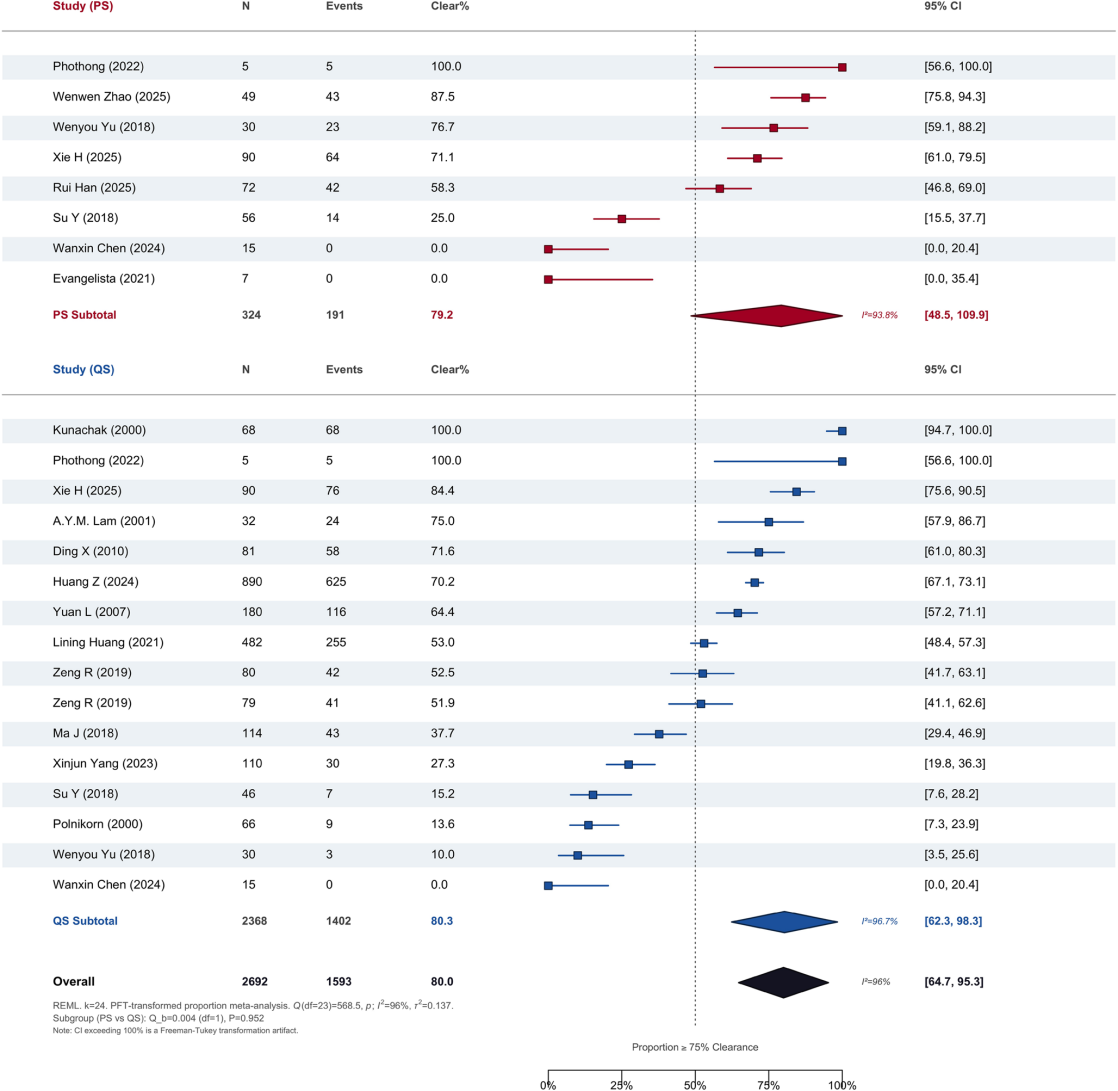
Forest plot of ≥ 75% clearance: PS vs. QS lasers. Squares, individual study estimates (size ∝ inverse-variance weight); diamonds, pooled estimates (random-effects model)

Twenty-four treatment arms (PS, 8; QS, 16) entered the ≥ 75% analysis. Under the primary GLMM analysis, pooled ≥ 75% clearance was 47.8% (95% CI 13.4–84.4) for PS and 53.1% (95% CI 30.3–74.6) for QS lasers (*P* = 0.95). As a sensitivity analysis using the Freeman-Tukey double arcsine (PFT) transformation, pooled ≥ 75% clearance was 79.2% (95% CI 48.5–109.9; I² = 93.8%; 95% PI, − 28.3–186.8%) for PS arms and 80.3% (95% CI 62.3–98.3; I² = 96.7%; 95% PI, 1.4–159.3%) for QS arms, with no significant between-group difference (Q = 0.004, *P* = 0.95) (Fig. 2). Because boundary proportions (0% and 100%) were present, the PFT upper CI for PS exceeded 100%—a recognized transformation artifact—whereas the GLMM estimate, bounded between 0% and 100%, provides a more interpretable result. Both estimation methods consistently showed no significant PS-versus-QS difference (Table 2). Notably, the 95% prediction intervals from the PFT model were extremely wide (PS: −28.3–186.8%; QS: 1.4–159.3%), indicating that future individual studies could produce clearance estimates spanning nearly the entire possible range—a statistical expression of the profound heterogeneity that fundamentally limits the precision of any pooled point estimate. Studies of 1064-nm QS Nd: YAG [14] and 755-nm QS alexandrite lasers constituted the majority of the QS evidence base. In an exploratory wavelength-stratified analysis restricted to QS lasers, 1064-nm arms showed numerically higher pooled clearance than 755-nm arms at both thresholds (≥ 75%: 60.5% vs. 47.2%; ≥90%: 55.6% vs. 37.3%; Supplementary Table S3). No formal between-group statistical testing was performed for this exploratory analysis. The numerically higher clearance with 1064-nm compared to 755-nm QS lasers is biologically plausible—longer wavelengths penetrate deeper into the mid-dermis where ABNOM melanocytes reside—but this post-hoc observation remains speculative and requires prospective verification. Notably, whether the included 1064-nm and 755-nm QS studies used comparable treatment endpoints (e.g., immediate whitening vs. fixed-parameter protocols) could not be determined from the reported data, representing a potential unmeasured confounder of this wavelength-stratified comparison. Of the included studies, PS lasers were predominantly 755 nm; 730-nm and 1064-nm devices were each used in two studies, with a single study at 670 nm. QS lasers spanned 694 to 1064 nm, with 1064-nm Nd: YAG being the most common wavelength. Within-PS wavelength effects have also been reported: a single small RCT (Chen et al., 2024; Ref 7) compared 730-nm versus 1064-nm PS and found that neither arm reached 75% clearance after a single session; sub-threshold differences cannot be excluded. This underscores that wavelength effects may exist within, not only between, laser platforms, though within-platform comparative evidence remains extremely limited.

**Table 2:** Sensitivity analyses for ≥ 75% clearance

Analysis	*n*	PS ≥ 75% (95% CI)	QS ≥ 75% (95% CI)	*P*
Primary (GLMM)	24	47.8% (13.4–84.4)	53.1% (30.3–74.6)	0.95
SA0: PFT transformation*	24	79.2% (48.5–109.9)	80.3% (62.3–98.3)	0.95
SA1: Low ROB	24	79.2% (48.5–109.9)	80.3% (62.3–98.3)	> 0.05
SA2: *N* ≥ 20	19	93.0% (70.6–115.3)	81.8% (64.7–98.8)	> 0.05
SA3: Exclude mixed λ	22	79.2% (48.5–109.9)	78.0% (57.5–98.5)	> 0.05
SA4: Exclude 0% effect	22	88.7% (61.1–116.3)	84.4% (67.4–101.4)	> 0.05
≥ 90% Clearance	28	74.5% (22.7–126.3)†	77.4% (67.5–87.3)	—

*PFT* Freeman-Tukey double arcsine, *GLMM* generalized linear mixed model, *ROB* risk of bias. *Upper confidence bound exceeding 100% is a recognized artifact of the Freeman-Tukey transformation near boundary proportions; the GLMM estimate provides a bounded alternative. †PS PFT estimate for ≥ 90% clearance; upper CI > 100% reflects transformation artifact. Formal between-group comparison not meaningful (3 PS arms only). Subgroup Q-tests for SA1–SA4 yielded uniformly non-significant between-group differences (*P* > 0.05) on the PFT scale

### Dose-response meta-regression

**Fig. 3:**
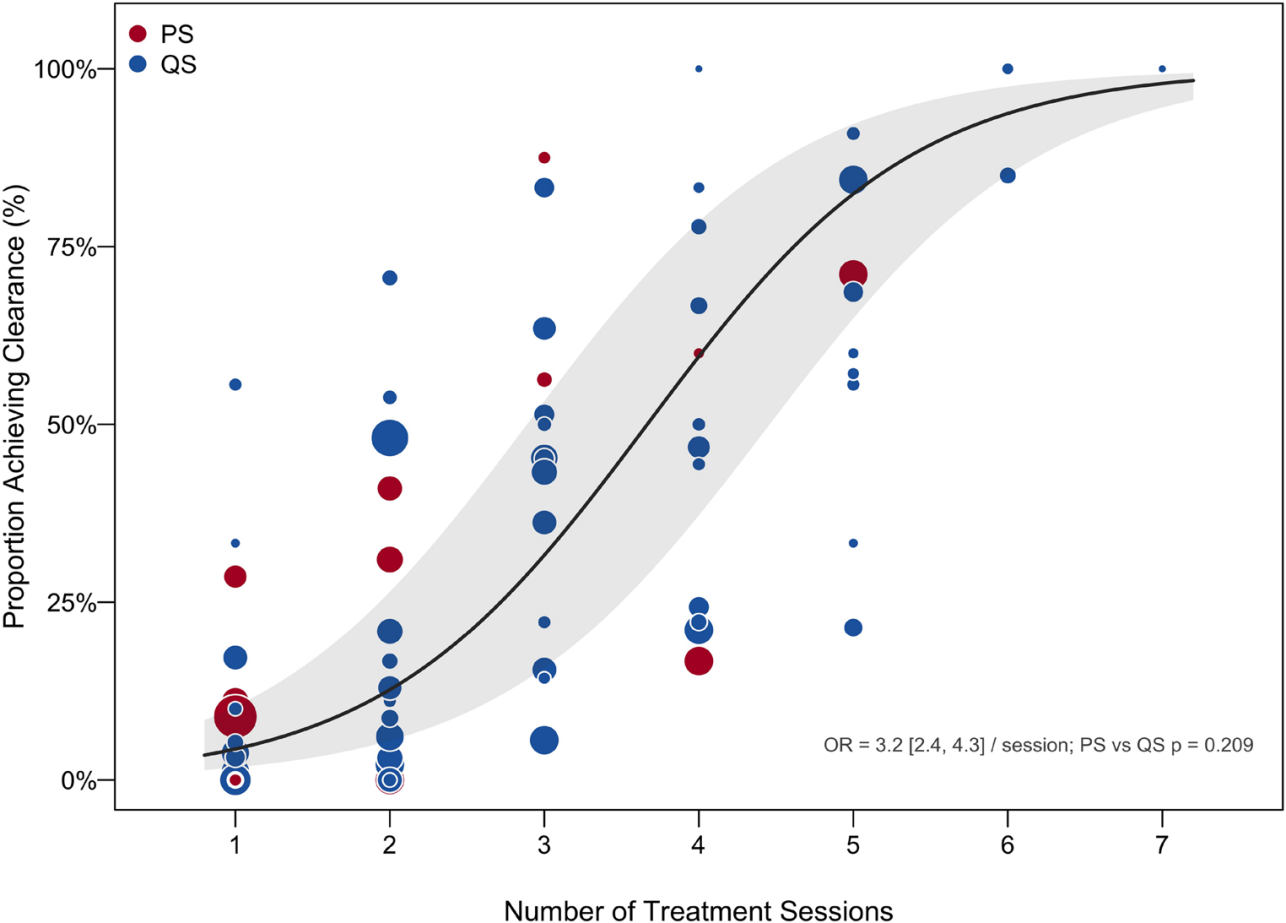
Dose-response meta-regression (threshold-adjusted). Point size ∝ √N. Triangles, PS; circles, QS. Solid line, fitted model; shading, 95% CI. OR/session = 3.2 (95% CI 2.4–4.3; cluster-robust *P* < 0.001). Random-slopes sensitivity (≥ 75% only): OR = 3.6 (95% CI 3.0–4.4)

Seventy-five observations from 18 treatment arms (PS, 5; QS, 13) entered the dose-response meta-regression (Fig. 3), with threshold level (≥ 75% vs. ≥90%) included as a covariate to prevent confounding by outcome definition. In the primary model, each additional treatment session was associated with an odds ratio of 3.2 (95% CI 2.4–4.3; *P* < 0.001) for achieving ≥ 75% clearance, based on cluster-robust standard errors accounting for within-study serial correlation. The threshold offset was not significant (logit coefficient = 0.71, *P* = 0.29), indicating that threshold level did not systematically bias the dose-response estimate. Adding laser type to the model did not improve fit: the PS-versus-QS level difference was not significant (robust *P* = 0.21). A random-slopes sensitivity model restricted to ≥ 75% data converged and yielded a consistent estimate (OR 3.6; 95% CI 3.0–4.4; *P* < 0.001). A quadratic term for session number was not significant (*P* = 0.33) and provided negligible improvement in model fit (linear model AIC = 279.3; quadratic model AIC = 279.4; ΔAIC < 2), supporting the linearity assumption over the observed 1- to 7-session range. Cluster-robust standard errors widened the session coefficient confidence interval by approximately 25% compared with model-based standard errors, confirming the importance of accounting for within-study serial correlation. An exploratory threshold-by-session interaction was not significant (*P* = 0.29), indicating that the dose-response slope did not differ meaningfully between the two outcome thresholds. The relationship was monotonic across the observed 1- to 7-session range, but should not be extrapolated beyond seven sessions, as the model is based on aggregated study-level data and cannot capture individual treatment-interval variability. Treatment intervals varied considerably across the included case series (4–12 weeks), representing an unmeasured confounder that may affect the observed session-response gradient. Moreover, laser fluence, spot size, and treatment endpoint (e.g., immediate whitening vs. fixed parameters) were inconsistently reported across studies, precluding analysis of these critical technical determinants of efficacy and safety; shorter intervals could produce faster apparent clearance per session, while longer intervals allow more time for pigment clearance between treatments.

###  ≥ 90% clearance

Twenty-eight treatment arms (PS, 3; QS, 25) from 24 unique studies provided ≥ 90% clearance data. With only three PS arms available (PS pooled ≥ 90%: 74.5%; 95% CI 22.7–126.3; I² = 90.9%; PFT estimate; the upper confidence bound exceeding 100% is a recognized transformation artifact), a formal between-group comparison would be uninterpretable. The pooled QS ≥ 90% clearance rate under GLMM was 77.4% (95% CI 67.5–87.3).

### Risk of bias and sensitivity

ROB assessment (Figures S1, S2) classified 38 of 40 studies as Low risk (95%) and 2 as Moderate risk (5%; see ROB_assessment.csv in Supplementary Materials). The two moderate-risk studies (Liu et al., 2015; Ge et al., 2014) contributed only to the ≥ 90% pool, so all 24 arms in the ≥ 75% analysis were Low risk; consequently, SA1 (exclusion of moderate-ROB studies) yielded results identical to SA0 (Table 2). Across all six sensitivity analyses, the absence of a significant PS-versus-QS difference was preserved (Table 2). In sensitivity analyses spanning both estimation methods, the pooled PS PFT estimate ranged from 79.2% (all arms) to 93.0% (SA2, *N* ≥ 20); the corresponding GLMM estimates ranged from 47.8% to 88.7%. This finding indicates that the pooled efficacy estimate of picosecond (PS) lasers is highly sensitive to two treatment arms from one study with zero clearance outcomes (Chen et al., 2024, 730-nm PS arm, *N* = 15; Chen et al., 2024, 1064-nm PS arm, *N* = 15). Excluding them (SA4) elevated the PS estimate from 47.8% (primary GLMM) to 88.7% (sensitivity PFT), though the upper confidence limit of 116.3% reflects the Freeman-Tukey transformation artifact. This fragility precludes any confident characterization of PS efficacy (Figure S3). QS estimates were comparatively stable, ranging from 53.1% to 84.4%.

### Publication bias

**Fig. 4:**
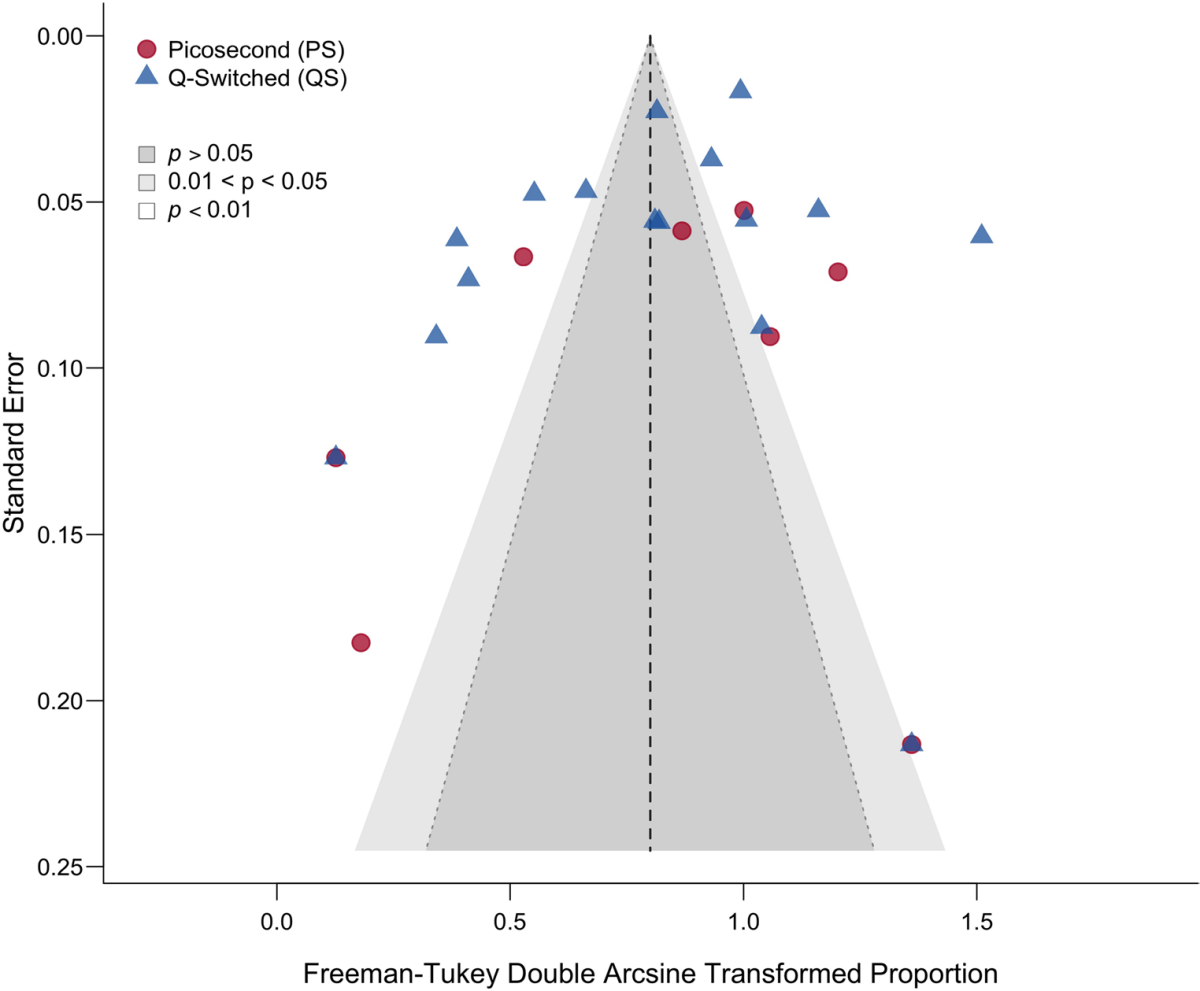
Contour-enhanced funnel plot for publication bias. Contour lines represent significance levels (light grey: *P* > 0.05; medium grey: 0.01 < *P* < 0.05; dark grey: *P* < 0.01). Peters test, *P* = 0.237

The funnel plot showed no gross asymmetry (Fig. 4). Peters’ test was not significant (t = − 1.228, *P* = 0.237). This analysis was conducted on the Freeman-Tukey double arcsine (PFT) transformed scale; the upper confidence limit exceeding 100% reflects a well-documented artifact of this transformation. Trim-and-fill analysis imputed two studies and produced an adjusted pooled estimate of 86.4% (95% CI 69.7–103.0).

### Certainty of evidence (GRADE)

Starting from “very low” (predominantly single-arm case series), the evidence for ≥ 75% clearance was rated across five domains (Table 3). The evidence was downgraded one level each for risk of bias and inconsistency (I² > 90%; heterogeneous outcome grading definitions), and this was partially offset by upgrading for a dose-response gradient (+ 1; exploratory). The final GRADE rating was **VERY LOW**. Because the dose-response gradient was derived from the same observational evidence base that was downgraded for risk of bias and inconsistency, the upgrade is exploratory and does not substantively alter the overall evidence certainty. For ≥ 90% clearance, sparse PS data and wider CIs precluded upgrading for dose-response; the rating remained **VERY LOW**.

**Table 3:** GRADE evidence profile for ≥ 75% clearance (PS vs. QS lasers)

Domain	Judgment	Action
Risk of bias	Serious (predominantly single-arm series)	−1
Inconsistency	Serious (I² > 90%)	−1
Indirectness	Not serious	0
Imprecision	Not serious (CIs informative and consistent)	0
Publication bias	Not detected (Peters *P* = 0.237)	0
Large effect	Not applicable (no large relative effect for PS-vs-QS contrast)	0
Dose-response gradient	Present (monotonic; robust to serial corr.)	+ 1
Overall certainty		VERY LOW

The dose-response trend was observed across independently grouped session-level data, distinct from the sources of downgrading (study design limitations and between-study heterogeneity). Starting level: Very low (observational, single-arm). ≥90% clearance remained VERY LOW (no upgrading)

## Discussion

### Principal findings

These data challenge the prevailing narrative of picosecond superiority in ABNOM treatment. Although PS lasers offer theoretical advantages—shorter pulse durations and stronger photomechanical disruption—these do not translate into a statistically significant efficacy gain in practice. As an exploratory finding, the clinical message tentatively suggests that completing four to six treatment sessions may matter substantially more than which laser platform delivers them. However, this dose-response finding must be interpreted with caution: the model operates on aggregated study-level data and cannot distinguish individual patient trajectories from ecological-level associations. Unmeasured confounders—including Fitzpatrick skin type, lesion severity, laser fluence, and patient self-selection (those adherent to seven sessions may represent inherently better responders)—preclude causal inference. The session-response gradient should therefore be regarded as hypothesis-generating rather than prescriptive.

### Comparison with the literature

The sole prior ABNOM-specific review, by Kaur et al. (2020), was qualitative and covered 19 studies without pooled estimates [4]. Individual comparative studies have noted higher early-session clearance with PS lasers, with the gap narrowing as session counts increase [6, 9]. Our meta-regression formalizes that observation: the PS and QS clearance followed parallel dose-response curves under a common-slope model (robust *P* = 0.21 for level difference). From the broader pigmentary laser literature, treatment session count consistently emerges as the strongest predictor of clearance [15].

### Clinical implications

Patients able to complete four to six treatment sessions can expect comparable outcomes from PS or QS lasers; platform choice may therefore be guided by device availability, cost, and patient preference rather than by purported efficacy differences. We propose a pragmatic framework: (1) Based on individual studies included in this review (not pooled in the meta-analysis), PS lasers may yield numerically faster early improvement, but this gap appears to narrow with additional sessions, though this observation is based on limited comparative data and requires confirmation; (2) QS lasers represent an equally effective and often more accessible alternative; and (3) the key determinant of clearance is treatment adherence and session completion, not pulse duration (predicted clearance rates by session number are provided in Supplementary Table S2). Emerging safety data further support this framework: picosecond lasers are associated with lower rates of post-inflammatory hyperpigmentation [16], a consideration relevant to Asian patients who constitute the majority of the ABNOM population.

### Safety considerations

Post-inflammatory hyperpigmentation (PIH) was registered as a co-primary safety endpoint (PROSPERO: CRD420261404917) but could not be quantitatively synthesized because adverse event reporting was highly heterogeneous across studies. Definitions of PIH varied (transient vs. persistent), denominator specification was inconsistent (per lesion, per patient, or per session), and many studies omitted safety data entirely. Among the 40 included studies, approximately 15 reported PIH rates qualitatively; crude rates ranged from 0% to 16% for QS lasers and 0% to 8% for PS lasers in those providing extractable data (e.g., > 10% transient PIH with 1064-nm QS in Asian skin [17]; 0% with 755-nm PS [6]). These figures should be interpreted with extreme caution given the inconsistent reporting; they should not be compared across studies or cited as true PIH incidence rates for any specific laser. Transient erythema and edema were near-universal but self-limiting. Scarring and permanent pigmentary changes were rarely reported (fewer than five cases across all studies). No eligible safety-focused subgroup analyses (e.g., by wavelength or Fitzpatrick skin type) could be performed. The absence of systematic safety reporting in the ABNOM laser literature represents a critical evidence gap; future studies should adopt standardized adverse event definitions—such as the Common Terminology Criteria for Adverse Events (CTCAE) or a dermatology-specific consensus instrument—with clear denominator specification. A recent meta-analysis of 21 randomized trials comparing picosecond and nanosecond lasers reported significantly lower rates of post-inflammatory hyperpigmentation (*P* = 0.02) and hypopigmentation (*P* = 0.002) with picosecond devices [16], consistent with the direction of safety signals observed across our included studies. In a split-lesion RCT directly comparing picosecond and Q-switched alexandrite lasers for nevus of Ota—a dermal melanocytosis closely related to ABNOM—Ge et al., 2020 [18] reported lower rates of PIH (26% vs. 34%) and hypopigmentation (21% vs. 47%) with the picosecond device [18]. Beyond pigmentary adverse events, comparative data indicate that immediate purpura, crusting, and blistering are more frequent with nanosecond devices, whereas picosecond treatment is typically associated with milder, shorter-lived local reactions [16, 18].

### Limitations

The evidence base is dominated by single-arm case series (30 of 40 studies). Most included studies were retrospective with non-consecutive patient enrollment, which carries a risk of selection bias and may systematically overestimate the pooled clearance rate. Heterogeneity was substantial (I² > 90%), reflecting genuine variation in treatment protocols, outcome grading systems, and patient demographics. Within-study serial correlation in the dose-response model was addressed with cluster-robust standard errors and confirmed by a random-slopes sensitivity model; both approaches yielded consistent estimates. Inter-rater agreement at screening fell below the registered target (κ = 0.33 vs. 0.75), attributable to the first reviewer’s intentionally inclusive strategy rather than unreliability. A minority of included studies defined clearance as > 75% rather than ≥ 75%; these were pooled under the ≥ 75% threshold, introducing a theoretical measurement inconsistency; however, given the inherent subjectivity of physician-assessed pigment clearance in clinical practice, this minor threshold discrepancy is unlikely to introduce meaningful bias. Safety outcomes, registered as co-primary endpoints, could not be quantitatively synthesized owing to inconsistent adverse-event reporting across studies. GRADE certainty was rated VERY LOW. Diagnostic criteria for ABNOM also varied across studies; most relied on clinical diagnosis alone, and differential diagnosis from dermal melasma or nevus of Ota was not consistently validated, which may have introduced additional population heterogeneity. PS data were sparse at the ≥ 90% threshold. Most studies enrolled East Asian populations, limiting generalizability to other ethnic groups. PS and QS wavelength distributions are detailed in Sect. 3.2. Two included studies—a split-face comparison of nanosecond and picosecond 1064-nm lasers (*N* = 5) [19] and a 670-nm PS case series (*N* = 22) [20]—used small samples; sensitivity analyses confirmed that their exclusion did not alter the primary conclusions. The Freeman-Tukey upper confidence bound exceeded 100% for the PS subgroup, a recognized artifact near boundary proportions. One study contributed per-session observations with *N* = 1 at certain time points, for which continuity correction was applied; these sparse data points had negligible influence on the fitted dose-response curve; however, the continuity correction of 0.5 for single-patient sessions represents a methodological limitation inherent to logit transformation of boundary proportions. The pooled ≥ 90% clearance rate numerically exceeded the ≥ 75% rate (Table 2), reflecting that the ≥ 90% pool drew on a different, more selective subset of studies with stricter inclusion criteria and more uniform treatment protocols; this does not represent a logical contradiction.

### Future directions

Heterogeneous outcome reporting across ABNOM laser studies hinders evidence synthesis. Future clinical studies should adopt standardized percentage-based clearance grading and unified adverse event assessment criteria. Large, multicenter randomized controlled trials with blinded outcome assessment and standardized treatment protocols are urgently needed to validate our findings.

## Conclusions

In this first quantitative meta-analysis of laser treatment for ABNOM, PS and QS lasers achieved clinically comparable clearance rates. Treatment session number—not laser type—showed a stronger association with clearance: each additional session approximately tripled the odds of achieving ≥ 75% clearance (OR 3.2; cluster-robust 95% CI 2.4–4.3). However, given the very low certainty of evidence, the predominance of single-arm data, and the extreme fragility of the pooled PS estimate (ranging from 47.8% to 88.7% depending on inclusion of two small-sample zero-clearance arms), the comparative efficacy of picosecond versus Q-switched lasers remains uncertain. The absence of a statistically significant difference does not establish equivalence; the PS evidence base remains underpowered. QS lasers, backed by a larger and more robust evidence base, represent the current most extensively studied modality. These pooled estimates may also partially reflect wavelength-related confounding, as deeper-penetrating 1064-nm QS lasers showed numerically higher clearance than 755-nm devices (Supplementary Table S3), while the PS evidence base predominantly comprises 755-nm wavelengths. These conclusions apply specifically to isolated ABNOM without concurrent melasma; the comparative efficacy profile may differ in patients with mixed pigmentary disorders. Importantly, the inconsistent reporting of safety outcomes precludes a valid risk-benefit comparison between laser modalities; any clinical recommendation must be weighed against this critical evidence gap. Clinical decisions should prioritize treatment adherence and session completion; QS lasers, supported by a more robust evidence base, remain the current evidence-supported standard, with PS lasers as an alternative.

## Supplementary Information

Below is the link to the electronic supplementary material.

Supplementary Material 1 (CSV 3.08 KB)

Supplementary Material 2 (DOCX 9.87 KB)

Supplementary Material 3 (DOCX 35.0 KB)

Supplementary Material 4 (DOCX 37.4 KB)

Supplementary Material 5 (DOCX 37.4 KB)

Supplementary Material 6 (DOCX 48.6 KB)

Supplementary Material 7 (DOCX 37.9 KB)

Supplementary Material 8 (DOCX 36.3 KB)

Supplementary Material 9 (DOCX 40.6 KB)

## Data Availability

All data are included in this article and its supplementary files. Analysis datasets and R scripts are available from the corresponding author upon reasonable request.

## Declarations

### **PROSPERO registration**

CRD420261404917.
