# Supplementary material for: Efficacy of Q-Switched and picosecond lasers for acquired bilateral nevus of Ota-like macules: a systematic review and exploratory dose-response meta-analysis
Source: Lasers Med Sci. 2026 Aug 6;41(1):180. doi: 10.1007/s10103-026-04982-6 (PMC13447452; doi:10.1007/s10103-026-04982-6)
Supplement: Supplementary file 1 [file 10103_2026_4982_MOESM2_ESM.docx]

**Supplementary Table S3. Wavelength-stratified clearance rates.**

| **≥75% Clearance** |  |  |  |  |
| --- | --- | --- | --- | --- |
| **Wavelength (nm)** | **Laser** | **PS arms (n)** | **QS arms (n)** | **Clearance rate** |
| 670 | PS | 1 | — | 0.0% (seq160, N=7) |
| 730 | PS | 2 | — | seq6: 58.3% (N=72); seq13-1: 0.0% (N=15) |
| 755 | PS | 4 | — | Pooled 65.0% [58.7–71.1] |
| 755 | QS | — | 6 | Pooled 47.2% [43.7–50.7] |
| 1064 | PS | 1 | — | 100.0% (seq22-1, N=5) |
| 1064 | QS | — | 9 | Pooled 60.5% [56.8–64.1] |
| Mixed | QS | — | 1 | 70.2% (seq12, N=890; 694/755/1064 nm) |

*Pooled estimates were computed using the Freeman-Tukey double arcsine transformation under a random-effects model for subgroups with ≥3 arms. PS, picosecond; QS, Q-switched. Mixed wavelengths were excluded from pooled analyses. The 1064-nm QS subgroup showed numerically higher pooled clearance than the 755-nm QS subgroup at both the ≥75% (60.5% vs. 47.2%) and ≥90% (55.6% vs. 37.3%) thresholds.*

| **≥90% Clearance** |  |  |  |  |
| --- | --- | --- | --- | --- |
| **Wavelength (nm)** | **Laser** | **PS arms (n)** | **QS arms (n)** | **Clearance rate** |
| 670 | PS | 1 | — | 0.0% (seq160, N=7) |
| 755 | PS | 2 | — | seq27: 60.0% (N=225); seq40-1: 80.0% (N=40) |
| 755 | QS | — | 4 | Pooled 37.3% [32.2–42.5] |
| 1064 | QS | — | 19 | Pooled 55.6% [52.9–58.3] |
| 752 | QS | — | 1 | seq136: 41.6% (N=202) |

*For the ≥90% threshold, no PS subgroup achieved ≥3 arms, precluding formal pooling.*
