# Supplementary material for: Efficacy of Q-Switched and picosecond lasers for acquired bilateral nevus of Ota-like macules: a systematic review and exploratory dose-response meta-analysis
Source: Lasers Med Sci. 2026 Aug 6;41(1):180. doi: 10.1007/s10103-026-04982-6 (PMC13447452; doi:10.1007/s10103-026-04982-6)
Supplement: Supplementary file 2 [file 10103_2026_4982_MOESM3_ESM.docx]

**Efficacy and Safety of Lasers for Hori's Nevus**

**(Acquired Bilateral Nevus of Ota-like Macules, ABNOM)**

**A Systematic Review and Meta-Analysis**

Study Protocol

Principal Investigator: Feng Li

Department of Dermatology, Changzhou Dean Hospital

Changzhou, Jiangsu, China

PROSPERO Registration ID: CRD420261404917

# **1. Introduction**

## **1.1 Background and Rationale**

Hori's nevus (Acquired Bilateral Nevus of Ota-like Macules, ABNOM), also known as acquired dermal melanocytosis (ADM) or nevus fusco-caeruleus zygomaticus, is a common acquired dermal pigmentary disorder predominantly affecting middle-aged Asian women. It is characterized by bilaterally symmetric, relatively well-demarcated brownish-blue to slate-gray macules distributed over the malar regions, nose, temples, and forehead. Unlike nevus of Ota, the melanocytes in ABNOM are typically located in the upper and mid dermis without mucosal involvement. Although benign, the condition is often associated with significant psychosocial burden and impairment in quality of life, creating strong demand for effective treatment.

The pathogenesis involves aberrant dermal melanocytes that fail to complete migration during embryogenesis. Laser therapy, operating through selective photothermolysis (nanosecond-domain) or photoacoustic effects (picosecond-domain), is the first-line treatment. Clinically available modalities include:

**Q-switched (nanosecond-domain) lasers:** Nd:YAG 1064 nm, alexandrite 755 nm, ruby 694 nm

**Picosecond-domain lasers:** alexandrite 755 nm, Nd:YAG 1064 nm, titanium sapphire 730 nm

**Combination regimens:** 532 nm + 1064 nm Nd:YAG, CO₂ + Q-switched ruby, etc.

The recent introduction of picosecond-domain lasers has shown promise for superior efficacy with reduced thermal damage, but their advantage over conventional Q-switched lasers remains contentious, with conflicting results across studies. Wang et al. (Dermatologic Therapy, 2020; PMID: 31714651) published the first systematic review of Hori's nevus management, including 19 studies, but performed only qualitative synthesis without quantitative meta-analysis. Since 2018, the picosecond laser literature has expanded rapidly, generating multiple high-quality studies—including at least two split-face randomized controlled trials (RCTs)—yielding a body of evidence now sufficient for quantitative synthesis.

**Identified research gaps:**

1. No quantitative meta-analysis on laser treatment for ABNOM has been published.

2. No evidence-based direct or indirect comparison of efficacy across laser modalities exists.

3. The clinical controversy of Q-switched versus picosecond-domain lasers lacks pooled comparative data.

4. Safety outcomes, particularly post-inflammatory hyperpigmentation (PIH) and melasma induction, have not been systematically assessed across laser types.

**Rationale:** This review addresses the critical need for evidence-based comparisons between all available laser modalities for ABNOM. By providing the first comprehensive quantitative synthesis using a rigorous three-tier evidence framework, this review will directly inform clinical decision-making for this common and treatment-challenging condition. One meta-analysis focused exclusively on 755-nm picosecond laser for nevus of Ota (Medicine, 2026; PMID: 41731817), a distinct disease entity; no equivalent synthesis exists for ABNOM.

# **2. Review Objectives**

**Primary objective:** To systematically evaluate and quantitatively compare the efficacy (excellent clearance >75%, good clearance >50%) of different laser modalities for treating ABNOM.

**Secondary objectives:**

. Compare safety profiles (PIH rate, post-laser pigmentary alteration, hypopigmentation, recurrence, erythema) across laser types.

. Explore sources of heterogeneity through prespecified subgroup analyses and meta-regression.

. Compare Q-switched and picosecond-domain lasers through both direct (RCT) and exploratory indirect comparisons.

**Exploratory objective:** Through stratified subgroup analyses and meta-regression, explore associations between treatment parameters (number of sessions, fluence) and clearance rates. Note: these analyses are exploratory and subject to confounding by indication.

# **3. Methods**

## **3.1 Study Design**

**Systematic review and meta-analysis.**

Registration: PROSPERO (International Prospective Register of Systematic Reviews).

Reporting standard: PRISMA 2020 statement.

## **3.2 PICO Framework**

| **Element** | **Description** |
| --- | --- |
| Population | Patients of any age, sex, or Fitzpatrick skin type with clinically diagnosed Hori's nevus/ABNOM/ADM, based on typical clinical presentation and bilateral malar distribution. Patients with concurrent active melasma are eligible only if the study explicitly states melasma exclusion criteria or provides separate ABNOM subgroup data. |
| Intervention | Laser treatment of any type: Q-switched Nd:YAG 1064 nm, Q-switched alexandrite 755 nm, Q-switched ruby 694 nm, picosecond alexandrite 755 nm, picosecond Nd:YAG 1064 nm, picosecond titanium sapphire 730 nm, and combination laser regimens. Studies using only routine post-procedure care (moisturizer, sunscreen) are classified as single-laser group. Studies using topical bleaching agents (hydroquinone, tretinoin, tranexamic acid, azelaic acid) or oral medications are classified as laser-drug combination and analyzed separately. |
| Comparison | Direct comparison via head-to-head RCTs; exploratory indirect comparison via subgroup analyses of pooled single-arm proportions across laser types. |
| Outcomes | See Section 3.3. |
| Study Design | RCTs (split-face or parallel-group), prospective/retrospective cohort studies, case series (N ≥ 5). |

## **3.3 Outcomes**

| **Category** | **Outcome** | **Definition** |
| --- | --- | --- |
| Primary Efficacy | Excellent clearance rate | Proportion of patients achieving >75% pigment clearance |
| Secondary Efficacy | Good clearance rate | Proportion achieving >50% pigment clearance |
|  | Complete clearance rate | Proportion achieving complete clearance |
|  | Mean planned sessions | Number of planned treatment sessions (not actually received) |
| Primary Safety | PIH rate | Post-inflammatory hyperpigmentation incidence |
|  | Post-laser pigmentary alteration | Pigmentary changes including melasma induction/exacerbation (per original study definitions) |
| Secondary Safety | Hypopigmentation rate | Post-laser hypopigmentation incidence |
|  | Recurrence rate | Pigment recurrence during follow-up (extracted with recurrence definition and follow-up duration) |
|  | Erythema rate | Transient post-procedure erythema incidence |

## **3.4 Inclusion Criteria**

1. Patients clinically diagnosed with Hori's nevus/ABNOM based on typical presentation and distribution (excluding patients with concurrent active melasma or unclear diagnosis, unless separate ABNOM data provided).

2. Laser treatment of any type, wavelength, or parameter.

3. Reporting at least one of the prespecified outcomes.

4. Study types: RCTs (split-face or parallel-group), prospective/retrospective cohort studies, case series (N ≥ 5).

5. Language: no restrictions (Chinese, English, Korean, Japanese, and others).

6. Publication status: peer-reviewed literature (including Epub ahead of print).

7. Studies must explicitly exclude patients with concurrent active melasma or provide separate ABNOM subgroup data. Studies without clear melasma exclusion criteria will be classified as having uncertain diagnostic criteria and assessed in sensitivity analyses.

## **3.5 Exclusion Criteria**

1. Case reports (N < 5).

2. Reviews, editorials, letters, conference abstracts without primary data.

3. Nevus of Ota (congenital, unilateral) unless ABNOM subgroup data can be separately extracted.

4. Non-laser treatments (cryotherapy, chemical peeling, surgery).

5. Animal studies, in vitro studies.

6. Duplicate publications (only the most complete dataset included).

7. Studies without accessible English abstract whose full text cannot be translated adequately.

# **4. Search Strategy**

## **4.1 Databases**

| **Database** | **Coverage** | **Access** |
| --- | --- | --- |
| PubMed/MEDLINE | English mainstream | Free |
| Embase (via Ovid) | European & pharmacological | Temporary account |
| Cochrane Library (CENTRAL) | RCTs + systematic reviews | Free |
| Web of Science Core Collection | Multidisciplinary + citation tracking | Institutional |
| CNKI | Chinese literature | Institutional |
| Wanfang Data | Chinese literature | Institutional |
| CNKI/CDMD Dissertations | Chinese gray literature | Institutional |
| KoreaMed | Korean literature | Free |
| KCI (Korean Citation Index) | Korean literature | Free |
| J-STAGE | Japanese literature | Free |
| Google Scholar | Gray literature supplement | Free (first 100 results) |

**Note:** Embase accessed via temporary institutional account. China Biology Medicine disc (CBM) excluded as CNKI + Wanfang cover >95% of Chinese literature.

## **4.2 Search Strategy (PubMed Example)**

#1 Disease (high sensitivity):

"Hori nevus"[tiab] OR "Hori's nevus"[tiab] OR "nevus of Hori"[tiab] OR "acquired bilateral nevus of Ota-like macules"[tiab] OR "acquired bilateral nevus of Ota like macules"[tiab] OR "ABNOM"[tiab] OR "ABNOMs"[tiab] OR "acquired dermal melanocytosis"[tiab] OR "acquired dermal melanocytoses"[tiab] OR "dermal melanocytosis"[tiab] OR "ADM"[tiab] OR "facial melanocytosis"[tiab] OR "acquired Ota-like"[tiab] OR "nevus fusco-caeruleus zygomaticus"[tiab]

#2 Intervention (high sensitivity):

"laser"[tiab] OR "lasers"[tiab] OR "Q-switched"[tiab] OR "Q switched"[tiab] OR "picosecond"[tiab] OR "nanosecond"[tiab] OR "Nd:YAG"[tiab] OR "Nd YAG"[tiab] OR "alexandrite"[tiab] OR "ruby"[tiab] OR "titanium sapphire"[tiab] OR "IPL"[tiab] OR "intense pulsed light"[tiab] OR "low fluence"[tiab] OR "laser toning"[tiab] OR "photothermolysis"[tiab]

#3 = #1 AND #2

Search strategies for Embase, WoS, and regional databases will be adapted accordingly. No language, date, or publication type restrictions applied.

## **4.3 Search Dates**

From database inception to June 2026. Literature screening completed within one month of the final search date.

## **4.4 Supplementary Searches**

. Reference list screening of included studies and relevant reviews.

. Forward and backward citation tracking.

. Google Scholar first 100 results.

. CNKI and Wanfang dissertation databases for unpublished Chinese studies.

. ClinicalTrials.gov and Chinese Clinical Trial Registry (ChiCTR) for registered but unpublished trials.

**5. Study Selection and Data Extraction**

## **5.1 Screening Process**

Database search → Deduplication → Title/abstract screening → Full-text screening → Final inclusion.

Reference management: EndNote / Zotero. After deduplication, two reviewers independently screen in duplicate.

Disagreements resolved by discussion or third reviewer arbitration.

Exclusion reasons recorded at full-text screening stage. PRISMA 2020 flow diagram generated.

## **5.2 Data Extraction Form**

Standardized extraction form (Excel), covering the following domains:

**Study characteristics:** first author, publication year, country, study design (RCT/prospective/retrospective), single/multicenter.

**Patient characteristics:** sample size, age (mean ± SD or range), sex ratio, Fitzpatrick skin type, diagnostic criteria, disease duration, baseline severity.

**Intervention details:** laser type, wavelength, pulse mode, device model, fluence (J/cm²), spot size (mm), pulse frequency (Hz), pulse width, number of sessions, treatment interval, cooling method, adjunctive therapy.

**Efficacy assessment:** assessment method (blinded/unblinded, number of independent evaluators, standardized photography), clearance grading criteria with citation, counts for >75%, >50%, and complete clearance, assessment quality grade, mean number of planned sessions, last follow-up time.

**Safety outcomes:** PIH events (n/N), severity, duration; post-laser pigmentary alteration events (including melasma induction/exacerbation, with the study’s operative definition or marked “Unclear” if undefined); hypopigmentation, recurrence, other adverse events. When extracting pigmentary alteration data, we will attempt to distinguish transient PIH from persistent melasma-like changes; if the study does not differentiate, this is recorded as-is without forced reclassification.

**Risk of bias domain:** individual items from relevant assessment tool, numbers and reasons for loss to follow-up/withdrawal.

## **5.3 Non-English Literature**

. Prioritize data extraction from English abstracts and standard tables.

. Where abstract data are insufficient, full-text translation using DeepL, Google Translate (and Papago for Korean).

. Studies where translation fails to yield key data will be excluded and documented in the PRISMA flowchart.

**Limitation acknowledged:** Non-English literature will be screened and translated as needed, with potential translation limitations documented in the Discussion.

## **5.4 Inter-Rater Reliability**

. Prior to formal screening, two reviewers will independently pilot-test 10 randomly selected articles to calibrate inclusion/exclusion criteria and data extraction form understanding.

. Title/abstract screening: Cohen’s Kappa ≥ 0.75. Recalibration if below threshold.

. Full-text screening: Cohen’s Kappa ≥ 0.75.

. Continuous variable extraction: Intraclass Correlation Coefficient (ICC) ≥ 0.80.

. All disagreements resolved by discussion or third reviewer, documented in PRISMA flowchart.

**6. Quality Assessment**

## **6.1 Risk of Bias**

| **Study Type** | **Assessment Tool** |
| --- | --- |
| RCTs | Cochrane Risk of Bias 2.0 (RoB 2) |
| Non-randomized comparative | ROBINS-I |
| Case series | JBI Checklist for Case Series (Munn et al., 2014) |
| Retrospective cohorts | Newcastle-Ottawa Scale (NOS) |

**Key domains emphasized:**

. JBI Checklist: consecutive inclusion, diagnostic consistency, assessment objectivity (blinding, standardized photography, independent evaluator), follow-up completeness.

. Cochrane RoB 2: outcome measurement bias domain, given that most outcomes rely on subjective assessment.

## **6.2 GRADE Certainty Assessment**

Applying the GRADE framework (Grading of Recommendations, Assessment, Development, and Evaluation):

Starting level: For RCTs, the starting certainty is 'high', but we will pre-emptively downgrade one level to 'moderate' due to pervasive risk of outcome measurement bias (reliance on non-blinded PGA). For single-arm studies, the starting certainty is 'low'.

. Downgrade factors: risk of bias (especially non-blinded, non-independent assessment), inconsistency, indirectness, imprecision, publication bias.

. Risk of bias: downgrade 2 levels for high risk, 1 level for moderate risk.

. Imprecision: downgrade 1 level if 95% CI spans clinically significant benefit and harm, or total sample size < 300.

. Upgrade factors: large effect size, dose-response gradient, residual confounding likely to underestimate effect.

**Special note:** For outcomes assessed predominantly by non-blinded Physician Global Assessment (PGA), the risk of bias domain will be routinely downgraded.

# **7. Statistical Analysis**

## **7.1 Software**

**Primary:** R 4.x (metafor, meta, robumeta packages).

**Secondary:** Stata 18 (MSTA module), RevMan 5.4.

## **7.2 Effect Measures**

**Proportions:** Freeman-Tukey double arcsine transformed pooled proportion, back-transformed to original scale with 95% CI.

**Continuous:** Weighted or standardized mean difference (WMD/SMD) with 95% CI.

**RCT direct comparison:** Risk ratio (RR) or odds ratio (OR) with 95% CI, pooled if ≥ 2 RCTs share a common comparator arm.

## **7.3 Pooling Model**

All primary analyses use random-effects models (DerSimonian-Laird or REML estimation). Fixed-effect sensitivity analyses reported when I² < 30% and clinical homogeneity is adequate.

**Operational definition of clinical homogeneity:**

. Patient characteristics: Fitzpatrick type predominantly III–IV, consistent diagnostic criteria.

. Intervention characteristics: identical laser type (wavelength, pulse mode), treatment regimen (sessions, interval) within comparable ranges.

. Outcome assessment: consistent clearance grading criteria and evaluation methods.

When the above characteristics differ substantially between studies, random-effects models will be prioritized even if statistical heterogeneity is low (I² < 30%).

## **7.4 Heterogeneity Assessment**

. Cochran’s Q test + I² statistic.

. I² thresholds: < 25% (low), 25– 50% (moderate), 50– 75% (substantial), > 75% (high).

. Exploration: subgroup analyses and meta-regression.

**High-heterogeneity rule:** If I² > 75% in a subgroup and heterogeneity sources cannot be identified through subgroup or meta-regression analysis, quantitative pooling will be deferred; only descriptive summary will be reported, with explanation in the Discussion.

## **7.5 Evidence Hierarchy and Subgroup Analyses**

**Level 1 (Highest evidence) — RCT direct comparison:** For ≥ 2 head-to-head RCTs sharing a common comparator arm, RR/OR will be pooled via meta-analysis. If comparator arms differ, individual effect sizes will be presented descriptively with 95% CI.

**Level 2 (Exploratory) — Single-arm pooled proportions:** All single-arm studies included, pooled by laser type. Between-subgroup comparisons are exploratory; severe confounding exists. Language: “suggested,” “appeared” (not “demonstrated,” “proved”).

**Level 3 (Safety) — Safety outcomes independent pooling:** Safety outcomes pooled and presented by laser type.

**Sparse data rule:** Subgroups with < 3 studies will receive descriptive summary only, not quantitative meta-analysis.

| **Subgroup Variable** | **Categories** | **Purpose** |
| --- | --- | --- |
| Laser type | QS Nd:YAG / QS Alex / QS Ruby / PS Alex / PS Nd:YAG / PS 730nm / Combination | Compare efficacy across laser modalities |
| Wavelength | 1064 / 755 / 694 / 730 nm | Penetration depth effects |
| Pulse mode | Q-switched vs Picosecond-domain | Core clinical question |
| Number of sessions | ≤3 / 4–6 / >6 / unreported | Stratified pooling (not continuous regression) |
| Assessment quality | Low bias vs High/unclear bias | Anticipated largest heterogeneity source |
| Study design | RCT / Prospective / Retrospective | Design bias detection |
| Publication era | ≤2010 / 2011–2018 / 2019–2026 | Technology iteration effect |
| Fitzpatrick type | III–IV / V–VI | PIH risk stratification |
| Geographic region | East Asia vs Southeast/South Asia | Population differences |
| Recurrence follow-up | <1 yr / 1–2 yr / >2 yr | Follow-up duration impact |

**7.6 Meta-Regression (Exploratory; ≥ 10 studies per covariate)**

**️ Critical note:** Number of treatment sessions is excluded from continuous meta-regression due to reverse causality (patients with better clearance stop earlier; those with poor clearance continue). This variable is handled as a stratified subgroup variable. All meta-regression findings from single-arm data are exploratory and observational, subject to confounding by indication.

| **Covariate** | **Type** | **Purpose** |
| --- | --- | --- |
| Fluence | Continuous | Dose-response relationship with clearance |
| Sample size | Continuous | Small-study effects detection |
| Publication year | Continuous | Technology iteration / era confounding |
| Pulse mode | Categorical | Q-switched vs picosecond independent effect |
| Wavelength | Categorical | Penetration depth influence |
| Assessment quality | Categorical | Key covariate: evaluation bias |
| Study design | Categorical | Design-type effect on estimates |

**Multiple comparison statement:** Prespecified core subgroup analyses (pulse mode QS vs PS, laser type, wavelength) will not undergo multiplicity correction as they are hypothesis-testing by prior design. All exploratory subgroup analyses and meta-regression P-values will be interpreted conservatively, with their exploratory nature explicitly stated. The limitation of no formal multiplicity correction (e.g., Benjamini-Hochberg) will be acknowledged in the Discussion.

## **7.7 Non-Independent Data**

. Same patients, different lasers (split-face / self-controlled): extract only the primary laser group with more complete efficacy data.

. Different patients, different lasers: include separately but use robust variance estimation (robumeta package cluster correction) to account for within-study correlation.

Data nesting relationships will be clearly documented in the extraction form and PRISMA flowchart.

## **7.8 Publication Bias**

**Proportions:** Peters test (designed for binary outcomes) + funnel plot visual assessment. Traditional Egger test not applied to proportion data.

**Continuous:** Egger’s linear regression + funnel plot.

Requirement: ≥ 10 studies per subgroup for statistical testing (< 10: visual funnel plot assessment only, acknowledging low power). If publication bias is detected, trim-and-fill method will provide corrected pooled estimates in sensitivity analysis.

## **7.9 Sensitivity Analyses**

1. Leave-one-out analysis.

2. Exclude high risk-of-bias studies (JBI/NOS low-quality + RoB 2 high risk).

3. Exclude small studies (N < 10; N < 20).

4. Exclude early-era studies (≤ 2005; technology generation gap).

5. Exclude combination therapy studies.

6. Exclude non-East Asian population studies.

7. Exclude studies with non-blinded, non-independent assessment (detection of assessment bias).

8. Restrict to prospective studies only: exclude all retrospective case series to assess the impact of retrospective design on effect estimates.

9. Exclude studies with unclear diagnostic criteria: studies that do not explicitly exclude concurrent active melasma or fail to provide separate ABNOM subgroup data.

**8. Study Timeline**

| **Phase** | **Activity** | **Estimated Time** |
| --- | --- | --- |
| 1 | PROSPERO registration | June 2026, Week 1 |
| 2 | Formal search + deduplication + title/abstract screening | June 2026, Week 2–3 |
| 3 | Full-text screening + data extraction (dual independent) | June Wk4 – July Wk2 |
| 4 | Risk of bias assessment (dual independent) | July 2026, Week 3 |
| 5 | Statistical analysis (R/Stata) | July Wk4 – Aug Wk3 (4 weeks) |
| 6 | Manuscript drafting | Aug Wk4 – Sept Wk1 |
| 7 | Internal review + revision | Sept 2026, Week 2–3 |
| 8 | English editing + final formatting | Sept 2026, Week 4 |
| 9 | Journal submission | October 2026, Week 1 |

# **9. Target Journals**

| **Tier** | **Journal** | **IF** | **Notes** |
| --- | --- | --- | --- |
| Primary | Lasers in Surgery and Medicine | 2.3 | Free access; ASLMS flagship |
| Backup 1 | Dermatologic Surgery | 2.9 | Free access; ASDS flagship |
| Backup 2 | Journal of Cosmetic Dermatology | 2.3 | Free access; high acceptance |
| Safety | Lasers in Medical Science | 2.1 | Free access; Springer |

# **10. Innovation and Expected Contributions**

1. First quantitative meta-analysis of laser treatment for ABNOM, filling a recognized evidence gap.

2. First three-tier evidence framework (RCT direct + single-arm pooled + safety) applied to this clinical question.

3. Comprehensive multilingual search (Chinese, English, Korean, Japanese), minimizing language and regional publication bias.

4. Assessment quality subgroup analysis: first systematic investigation of evaluation bias impact on effect estimates.

5. Prudent safety outcome distinction (PIH vs laser-induced pigmentary alteration) with stratified reporting.

6. GRADE certainty ratings providing quantitative evidence hierarchy for clinical decision-making.

# **11. Study Team**

| **Role** | **Name** | **Responsibility** |
| --- | --- | --- |
| Principal Investigator | Feng Li | Study design, search, screening, data extraction, statistical analysis, manuscript writing |
| Second Author | TBD | Independent dual screening, data extraction, risk of bias assessment |
| Corresponding Author | TBD | Study supervision, quality control, manuscript review |

Statistical analysis code will be written and reviewed by the principal investigator. All code will be deposited in a public repository upon manuscript submission.

# **12. Ethics and Registration**

This study is a systematic review and meta-analysis of published aggregate data. It does not involve individual patient data or original human subject research and therefore does not require institutional review board (IRB) ethics approval. Only aggregate data from published literature will be extracted, involving no individual patient privacy information. If Chinese dissertation (gray literature) data are included, extraction and use will follow reasonable citation norms, with sources acknowledged. The study protocol will be prospectively registered with PROSPERO. Reporting will follow the PRISMA 2020 statement.

# **13. Key References**

1. Wang RF, et al. Therapeutic options for management of Hori's nevus: A systematic review. Dermatol Ther. 2020;33(1):e13172.

2. Ge Y, et al. A split-face RCT of alexandrite 755-nm picosecond vs nanosecond laser for ABNOM. J Am Acad Dermatol. 2018;79(3):479–485.

3. Yu N, et al. Comparison of 730-nm and 1064-nm picosecond lasers for ABNOM. J Cosmet Dermatol. 2024.

4. Chen J, et al. A retrospective study of 1064-nm QS Nd:YAG laser for ABNOM. Skin Res Technol. 2023;29(3):e13298.

5. Lee YB, et al. Association Between ABNOM Patients' Clinical Characteristics and Melasma Induction After Q-Switched Laser: A Decadal Study of 2675 Chinese Women. Lasers Surg Med. 2025.

6. Munn Z, et al. Methodological quality of case series studies: an introduction to the JBI critical appraisal tool. JBI Database System Rev Implement Rep. 2020.

7. Higgins JPT, et al. Cochrane Handbook for Systematic Reviews of Interventions. Version 6.4, 2023.

8. Page MJ, et al. The PRISMA 2020 statement: an updated guideline for reporting systematic reviews. BMJ. 2021;372:n71.
