# Supplementary material for: Efficacy of Q-Switched and picosecond lasers for acquired bilateral nevus of Ota-like macules: a systematic review and exploratory dose-response meta-analysis
Source: Lasers Med Sci. 2026 Aug 6;41(1):180. doi: 10.1007/s10103-026-04982-6 (PMC13447452; doi:10.1007/s10103-026-04982-6)
Supplement: Supplementary file 3 [file 10103_2026_4982_MOESM4_ESM.docx]

**Supplementary Table S4. Model diagnostics for all analyses.**
*tau², between-study variance; AIC, Akaike information criterion; BIC, Bayesian information criterion. GLMM models used logit link with random effects. REML models used restricted maximum likelihood estimation. Cluster-robust standard errors (18 clusters) were applied to all dose-response models. I² is not reported for GLMM and REML models because a universally accepted I² definition is not available for these frameworks; it is reported only for the PFT analysis where the conventional definition applies. AIC/BIC are not reported for GLMM proportion models because likelihood is not directly comparable across estimation methods and may be misleading given boundary proportions in some study arms.*

| **Model** | **Estimator** | **Effect estimate** | **tau²** | **AIC** | **BIC** | **Converged** | **Notes** |
| --- | --- | --- | --- | --- | --- | --- | --- |
| Proportion ≥75% (Primary) | GLMM | PS: 47.8% (13.4–84.4) QS: 53.1% (30.3–74.6) Between-group P = 0.95 | 3.96 | — | — | Yes | Logit link, random-effects |
| Proportion ≥75% (Sensitivity) | PFT | PS: 79.2% (48.5–109.9) QS: 80.3% (62.3–98.3) | — | — | — | Yes | Upper CI >100% is recognized PFT artifact. I²: PS 93.8%, QS 96.7% |
| Proportion ≥90% | GLMM | PS: 74.5% (22.7–126.3)† QS: 77.4% (67.5–87.3) | 1.27 | — | — | Yes | †PFT estimate; only 3 PS arms; comparison uninterpretable |
| Dose-response M1 (Linear) | REML | Session OR 3.2 (2.4–4.2) Threshold offset, P = 0.29 | 1.52 | 279.3 | 288.4 | Yes | Sessions + threshold; cluster-robust SE |
| Dose-response M2 (+Laser) | REML | Session OR 3.2 (2.4–4.3) Laser type (PS vs QS), P = 0.21 | 1.49 | 276.2 | 287.5 | Yes | Laser type not significant; robust SE |
| Dose-response M3 (Quadratic) | REML | Quadratic term, P = 0.33 ΔAIC vs linear = 0.1 | — | 279.4 | — | Yes | Supports linearity assumption |
| Session × Threshold | REML | Interaction, P = 0.29 | — | — | — | Yes | With laser_type; cluster-robust SE |
| Random-slopes (≥75% only) | REML | Session OR 3.6 (3.0–4.4) | σ² = 0 (boundary) | 102.2 | — | Yes | Boundary solution; model reduces to effective fixed-effects. REML/AIC reported from fitting stage |

Proportion meta-analysis Q-statistics (GLMM ge75: Q = 273.1, df = 23, P < 0.001; PFT ge75: Q = 568.5, df = 23, P < 0.001; GLMM ge90: Q = 221.1, df = 27, P < 0.001) are provided as supplementary diagnostic information. For the random-slopes model, the variance-covariance matrix converged to a boundary solution (σ² = 0); the session effect was estimated under study-level clustering without a random intercept component. This likely reflects sparse data (8 studies, 35 observations from the ge75 subset) rather than true homogeneity of intercepts. All CIs match the main text (±0.1 rounding).
