# Supplementary material for: Efficacy of Q-Switched and picosecond lasers for acquired bilateral nevus of Ota-like macules: a systematic review and exploratory dose-response meta-analysis
Source: Lasers Med Sci. 2026 Aug 6;41(1):180. doi: 10.1007/s10103-026-04982-6 (PMC13447452; doi:10.1007/s10103-026-04982-6)
Supplement: Supplementary file 4 [file 10103_2026_4982_MOESM5_ESM.docx]

**PRISMA 2020 Checklist**

| **Section/Topic** | **#** | **PRISMA 2020 Item** | **Reported on Page** |
| --- | --- | --- | --- |
| TITLE | 1 | Identify the report as a systematic review. | Title page |
| ABSTRACT | 2 | See the PRISMA 2020 for Abstracts checklist. | p.1 |
| INTRODUCTION |  |  |  |
| Rationale | 3 | Describe the rationale for the review in the context of existing knowledge. | pp.2-3 |
| Objectives | 4 | Provide an explicit statement of the objective(s) or question(s). | p.3 |
| METHODS |  |  |  |
| Eligibility criteria | 5 | Specify the inclusion and exclusion criteria. | pp.3-4 |
| Information sources | 6 | Specify all databases, registers, websites, etc. | p.4 / Appendix S3 |
| Search strategy | 7 | Present the full search strategies for all databases. | Appendix S3 |
| Selection process | 8 | Specify the methods used to decide whether a study met the inclusion criteria. | pp.4-5 |
| Data collection process | 9 | Specify the methods used to collect data from reports. | p.5 |
| Data items | 10 | List and define all outcomes for which data were sought. | p.5 |
| Risk of bias assessment | 11 | Specify the methods used to assess risk of bias. | pp.5-6 |
| Effect measures | 12 | Specify for each outcome the effect measure(s) used. | pp.6-7 |
| Synthesis methods | 13 | Describe the processes used to decide which studies were eligible for each synthesis. | pp.6-8 |
| Reporting bias assessment | 14 | Describe any methods used to assess risk of reporting bias. | p.7 |
| Certainty assessment | 15 | Describe the methods used to assess certainty in the body of evidence. | p.8 |
| RESULTS |  |  |  |
| Study selection | 16 | Describe the results of the search and selection process. | pp.8-9, Figure 1 |
| Study characteristics | 17 | Present characteristics of each included study. | Table 1 |
| Risk of bias in studies | 18 | Present assessments of risk of bias for each included study. | pp.12-13, Figures S1-S2 |
| Results of syntheses | 19 | Present the results of all statistical syntheses. | pp.9-13, Tables 2-3 |
| Reporting biases | 20 | Present assessments of risk of reporting biases. | p.13, Figure 4 |
| Certainty of evidence | 21 | Present assessments of certainty in the body of evidence. | p.13, Table 3 |
| DISCUSSION |  |  |  |
| Discussion | 22 | Provide a general interpretation of the results in the context of other evidence. | pp.14-16 |
|  | 23 | Discuss any limitations of the evidence included in the review. | pp.17-18 |
|  | 24 | Discuss any limitations of the review processes used. | pp.17-18 |
|  | 25 | Discuss implications of the results for practice, policy, and future research. | pp.15-17 |
| OTHER INFORMATION |  |  |  |
| Registration and protocol | 26 | Provide registration information for the review. | p.3 / PROSPERO CRD420261404917 |
| Support | 27 | Describe sources of financial or non-financial support. | Declarations |
| Competing interests | 28 | Declare any competing interests. | Declarations |
| Availability of data, code, etc. | 29 | Report which of the following are publicly available. | Declarations |

*From: Page MJ, McKenzie JE, Bossuyt PM, et al. The PRISMA 2020 statement: an updated guideline for reporting systematic reviews. BMJ 2021;372:n71. doi: 10.1136/bmj.n71*
