# Supplementary material for: Efficacy of Q-Switched and picosecond lasers for acquired bilateral nevus of Ota-like macules: a systematic review and exploratory dose-response meta-analysis
Source: Lasers Med Sci. 2026 Aug 6;41(1):180. doi: 10.1007/s10103-026-04982-6 (PMC13447452; doi:10.1007/s10103-026-04982-6)
Supplement: Supplementary file 5 [file 10103_2026_4982_MOESM6_ESM.docx]

**Supplementary Table S1. Studies excluded after full-text assessment and data extraction (n = 35).**

| **Study** | **Title** | **Reason for Exclusion** |
| --- | --- | --- |
| He Yanping, 2024 | Q开关694 nm激光联合美宝湿润烧伤膏治疗颧部褐青色痣的临床价值 | 694 nm fractional laser (non-standard Q-switched mode); clearance thresholds not extractable |
| Sun Haijuan, 2022 | 皮秒翠绿宝石激光治疗颧部褐青色痣的疗效和安全性 | Laser type unclassifiable (claimed picosecond but parameters consistent with nanosecond Q-switched; wavelength discrepancy); ≥75% clearance not extractable (grading straddles threshold) |
| Wang Tiange, 2022 | 合并黄褐斑的获得性太田痣样斑激光治疗后黄褐斑加重的临床分析 | All patients had concurrent melasma (mixed-pigmentation population); ABNOM-specific clearance data not isolable |
| Feng Hao, 2020 | 波长755nm皮秒激光治疗双侧获得性太田痣样斑合并黄褐斑的临床疗效 | All patients had concurrent melasma (mixed-pigmentation population) |
| Qiao Jiying, 2019 | 倍频Q开关Nd:YAG 532 nm激光治疗179例颧部褐青色痣回顾性分析 | 532 nm wavelength — targets epidermal melanin rather than dermal melanocytes; not part of the standard therapeutic armamentarium for dermal melanocytosis; no within-wavelength comparator available |
| SJ Lee, 2018 | The Efficacy of a Q-Switched 694-nm Ruby Fractional Laser for Acquired Bilateral Nevus of Ota-like Macules | Fractional (non-standard) Q-switched mode; clearance thresholds not extractable |
| B Wang, 2016 | Induction of melasma by 1064-nm Q-switched neodymium:yttrium-aluminum-garnet laser therapy for acquired bilateral nevus of Ota-like macules (Hori nevus): a study on related factors in the Chinese population | Mixed-pigmentation population (ABNOM with post-treatment melasma); no pre-treatment clearance data |
| Jiang Zixu, 2015 | 分析应用Q开关1064 nm激光小光斑低能量微爆破治疗褐青色痣的效果 | No quantifiable percentage-based clearance threshold reported |
| Li Tong, 2015 | Q开关1064nm激光治疗颧部褐青色痣82例疗效观察及分析 | No percentage-based clearance threshold criteria; reported only aggregate categories without defined cut-offs |
| Zhao Lina, 2014 | 调Q开关激光治疗面部褐青色痣的疗效观察 | Conference abstract; no quantifiable clearance grading system |
| Bao Jianyong, 2015 | Q开关1064nm激光小光斑低能量微爆破治疗褐青色痣的临床观察 | No quantifiable clearance threshold |
| Zhao Yanxia, 2014 | 调Q开关紫翠宝石激光治疗褐青色痣疗效观察 | Clearance grading threshold of 30% only; no ≥75% or ≥90% data extractable |
| Zhang Di, 2013 | MedLite C6 Q开关美肤激光治疗仪治疗颧部褐青色痣的临床观察 | Reported only "cure" rate (100% clearance); ≥75% or ≥90% threshold data not extractable |
| He Mei, 2007 | Q开关Nd:YAG激光治疗颧部褐青色痣疗效分析 | Mixed-pigmentation population (ABNOM with other facial pigmented lesions); ABNOM-specific data not isolable |
| Teng Xiaoping, 2007 | Q开关Nd:YAG激光治疗颧部褐青色痣与太田痣的临床分析 | Mixed-disease population (ABNOM plus nevus of Ota); ABNOM-specific clearance data not isolable |
| DH Suh, 2001 | Clinical use of the Q-switched Nd:YAG laser for the treatment of acquired bilateral nevus of Ota-like macules (ABNOM) in Koreans | Reported only 100% complete clearance rate; ≥75% and ≥90% threshold data not extractable from per-session reporting |
| Liao Xiaolin, 2021 | 分析Q开关1064nm激光小光斑低能量微爆破治疗褐青色痣的治疗价值 | No ≥75% or ≥90% clearance threshold data reported |
| Ma Gang, 2007 | Q开关Nd:YAG激光治疗颧部褐青色痣161例 | 532 nm wavelength — targets epidermal melanin rather than dermal melanocytes; not part of the standard therapeutic armamentarium for dermal melanocytosis; no within-wavelength comparator available |
| B Lee, 2004 | Comparison of characteristics of acquired bilateral nevus of Ota-like macules and nevus of Ota according to therapeutic response | Mixed-disease population (ABNOM plus nevus of Ota); ABNOM-specific clearance data not isolable |
| C Ungaksornpairote, 2020 | A Prospective, Split-Face, Randomized Study Comparing Picosecond to Q-Switched Nd:YAG Laser for Treatment of Epidermal and Dermal Pigmented Lesions in Asians | Clearance reported only as >50% improvement; ≥75% threshold data not extractable |
| Zhao Wenwen, 2025 | 1064 nm皮秒Nd:YAG激光治疗褐青色痣103例的回顾性研究 | No percentage-stratified clearance data; reported aggregate outcomes without defined percentage thresholds |
| Xiong Haojun, 2025 | 低能量模式和高能量模式Q开关1064 nm Nd:YAG激光治疗女性褐青色痣的疗效及不良反应对比观察：一项裂面分析 | Clearance assessed on a quartile scale without percentage-based cut-offs; no ≥75% or ≥90% threshold data |
| Van Lam Nguyen, 2025 | Associated Factors and Treatment Outcomes of Hori Nevus Treated with 1064-nm Q-Switched Nd:YAG Laser | Clearance grading system straddles extraction thresholds; grade boundaries incompatible with ≥75% or ≥90% mapping |
| Zhu Jian, 2020 | 调Q开关翠绿宝石激光治疗颧部褐青色痣患者的临床体会 | Clearance grading: marked improvement (70–94%) straddles 75% threshold; "cure" defined as 100% only — ≥90% subpopulation not extractable |
| Lan Ji, 2018 | 太田痣和获得性双侧太田痣样斑Q开关1064nm Nd:YAG激光治疗效果观察 | Clearance graded at 60% threshold only; no ≥75% or ≥90% data |
| Bai Li, 2011 | Q开关Nd:YAG激光治疗褐青色痣150例的疗效分析 | No independent per-session n/N data; clearance reported as aggregate proportions without session-level denominators |
| Tan Jun, 2010 | Q开关激光治疗双侧面部获得性太田痣样斑287例疗效分析 | "Cure" defined as complete pigment disappearance (100%) with no ≥90% subpopulation; ≥75% threshold not reported |
| Chen Lihua, 2009 | Q-开关翠绿宝石激光治疗颧部褐青色痣疗效观察 | No percentage-stratified clearance data; aggregate outcomes without defined thresholds |
| Li Chaohui, 2008 | Q开关激光治疗颧部褐青色痣980例疗效分析 | Aggregate proportions only; no independent per-session n/N data extractable |
| Chen Kang, 2008 | Q开关紫翠宝石激光治疗颧部褐青色痣116例 | Clearance thresholds incompatible: ≥95% does not map to ≥90%; 70–94% straddles 75% boundary |
| Teng Wen, 2008 | Q开关Nd:YAG激光治疗颧部褐青色痣280例 | Aggregate proportions without independent n/N; no session-level denominator data |
| Lü Rong, 2006 | 绿宝石激光治疗颧部褐青色痣54例 | "Cure" defined as complete clearance (100%); no ≥90% subpopulation; ≥75% threshold not extractable |
| Jia Yan, 2005 | Q开关Nd:YAG激光治疗颧颞部褐青色痣69例 | "Cure" defined as complete clearance (100%); no ≥90% subpopulation; ≥75% threshold not extractable |
| He Li, 2003 | Q开关紫翠宝石激光治疗颧部褐青色痣疗效分析 | Clearance graded at 30% boundary only; no ≥75% or ≥90% threshold data |
| Lin Zhongmei, 2017 | 不同波长调Q激光治疗颧部褐青色痣疗效分析 | No quantifiable ≥75% or ≥90% clearance thresholds; reported only qualitative efficacy categories |
