# Supplementary material for: Efficacy of Q-Switched and picosecond lasers for acquired bilateral nevus of Ota-like macules: a systematic review and exploratory dose-response meta-analysis
Source: Lasers Med Sci. 2026 Aug 6;41(1):180. doi: 10.1007/s10103-026-04982-6 (PMC13447452; doi:10.1007/s10103-026-04982-6)
Supplement: Supplementary file 6 [file 10103_2026_4982_MOESM7_ESM.docx]

**Appendix S3. Database Search Strategies**

All databases were searched from inception to May 27, 2026. No language restrictions were applied.

**1. PubMed**

Searched on May 26, 2026. Retrieved 154 records.

("Hori nevus"[tiab] OR "Hori's nevus"[tiab] OR "nevus of Hori"[tiab] OR "acquired bilateral nevus of Ota-like macules"[tiab] OR "acquired bilateral nevus of Ota like macules"[tiab] OR "ABNOM"[tiab] OR "ABNOMs"[tiab] OR "acquired dermal melanocytosis"[tiab] OR "acquired dermal melanocytoses"[tiab] OR "dermal melanocytosis"[tiab] OR "ADM"[tiab] OR "facial melanocytosis"[tiab] OR "acquired Ota-like"[tiab] OR "nevus fusco-caeruleus zygomaticus"[tiab]) AND ("laser"[tiab] OR "lasers"[tiab] OR "Q-switched"[tiab] OR "Q switched"[tiab] OR "picosecond"[tiab] OR "nanosecond"[tiab] OR "Nd:YAG"[tiab] OR "Nd YAG"[tiab] OR "alexandrite"[tiab] OR "ruby"[tiab] OR "titanium sapphire"[tiab] OR "IPL"[tiab] OR "intense pulsed light"[tiab] OR "low fluence"[tiab] OR "laser toning"[tiab] OR "photothermolysis"[tiab]) NOT "acellular dermal matrix"[tiab]

*Note: A NOT "acellular dermal matrix" clause was added to exclude false positives from the shared abbreviation "ADM".*

**2. Embase**

Searched on May 27, 2026 via Embase.com. Retrieved 215 records.

(hori nevus:ti,ab OR (hori NEAR/1 nevus):ti,ab OR nevus of hori:ti,ab OR acquired bilateral nevus of ota-like macules:ti,ab OR acquired bilateral nevus of ota like macules:ti,ab OR abnom:ti,ab OR abnoms:ti,ab OR acquired dermal melanocytosis:ti,ab OR acquired dermal melanocytoses:ti,ab OR dermal melanocytosis:ti,ab OR adm:ti,ab OR facial melanocytosis:ti,ab OR acquired ota-like:ti,ab OR nevus fusco-caeruleus zygomaticus:ti,ab) AND (laser:ti,ab OR lasers:ti,ab OR q-switched:ti,ab OR q switched:ti,ab OR picosecond:ti,ab OR nanosecond:ti,ab OR nd:yag:ti,ab OR nd yag:ti,ab OR alexandrite:ti,ab OR ruby:ti,ab OR titanium sapphire:ti,ab OR ipl:ti,ab OR intense pulsed light:ti,ab OR low fluence:ti,ab OR laser toning:ti,ab OR photothermolysis:ti,ab) NOT acellular dermal matrix:ti,ab

*Note: (hori NEAR/1 nevus) was used because Embase.com does not support double-apostrophe escaping for possessive forms within single-quoted phrases.*

**3. Cochrane CENTRAL**

Searched on May 26, 2026 via Cochrane Library (Trials). Retrieved 18 records.

```
#1  MeSH descriptor: [Laser Therapy] explode all trees
#2  MeSH descriptor: [Lasers, Solid-State] explode all trees
#3  (laser OR lasers OR Q-switched OR Q switched OR picosecond OR nanosecond OR Nd:YAG OR Nd YAG OR alexandrite OR ruby OR titanium sapphire OR IPL OR intense pulsed light OR low fluence OR laser toning OR photothermolysis):ti,ab,kw
#4  #1 OR #2 OR #3
#5  (Hori nevus OR Horis nevus OR nevus of Hori OR acquired bilateral nevus of Ota-like macules OR ABNOM OR ABNOMs OR acquired dermal melanocytosis OR acquired dermal melanocytoses OR dermal melanocytosis OR ADM OR facial melanocytosis OR acquired Ota-like OR nevus fusco-caeruleus zygomaticus):ti,ab,kw
#6  #4 AND #5
#7  #6 NOT acellular dermal matrix:ti,ab,kw
```

**4. Web of Science**

Searched via Web of Science Core Collection. Retrieved 285 records.

TS=("Hori nevus" OR "Horis nevus" OR "nevus of Hori" OR "acquired bilateral nevus of Ota-like macules" OR "acquired bilateral nevus of Ota like macules" OR ABNOM OR ABNOMs OR "acquired dermal melanocytosis" OR "acquired dermal melanocytoses" OR "dermal melanocytosis" OR ADM OR "facial melanocytosis" OR "acquired Ota-like" OR "nevus fusco-caeruleus zygomaticus") AND TS=(laser OR lasers OR "Q-switched" OR "Q switched" OR picosecond OR nanosecond OR "Nd:YAG" OR "Nd YAG" OR alexandrite OR ruby OR "titanium sapphire" OR IPL OR "intense pulsed light" OR "low fluence" OR "laser toning" OR photothermolysis) NOT TS="acellular dermal matrix"

**5. CNKI (China National Knowledge Infrastructure)**

Searched via cnki.net professional search. SU = subject (title + abstract + keywords).

SU=('Hori痣' + '褐青色痣' + '褐青色斑' + '获得性太田痣样斑' + '获得性双侧太田痣样斑' + 'ABNOM' + 'ADM' + '获得性真皮黑素细胞增多症' + '双侧太田痣样斑' + '颧部褐青色痣' + '颧部褐青色斑' + '面颈部褐青色痣' + '太田痣样斑') * ('激光' + '调Q' + 'Q开关' + '皮秒' + '纳秒' + '翠绿宝石' + '紫翠玉' + '红宝石' + '强脉冲光' + 'IPL' + '低能量' + '大光斑低能量' + '激光净肤' + '选择性光热解')

*CNKI syntax: + = OR, * = AND. Chinese disease terms: Hori痣, 褐青色痣, 获得性太田痣样斑, ADM, 颧部褐青色痣.*

**6. Wanfang Data**

Searched via wanfangdata.com.cn.

主题:(Hori痣 OR 褐青色痣 OR 褐青色斑 OR 获得性太田痣样斑 OR ABNOM OR ADM OR 获得性真皮黑素细胞增多症 OR 双侧太田痣样斑 OR 颧部褐青色痣 OR 颧部褐青色斑) AND 主题:(激光 OR 调Q OR Q开关 OR 皮秒 OR 纳秒 OR Nd:YAG OR 翠绿宝石 OR 紫翠玉 OR 红宝石 OR 强脉冲光 OR IPL OR 低能量 OR 激光净肤 OR 选择性光热解)

**7. CNKI/CDMD (Dissertations)**

Searched via CNKI dissertation database using the same strategy as CNKI (entry 5).

**8. KoreaMed, KCI, and KMBASE**

Searched using English and Korean-language terms. One record identified from KoreaMed.

English: Hori nevus OR ABNOM OR acquired dermal melanocytosis OR acquired bilateral nevus of Ota-like macules
Korean: 호리반 OR 후천성 진피 멜라닌세포증

**9. J-STAGE**

Searched via jstage.jst.go.jp. Retrieved 5 records.

(Hori nevus OR ABNOM OR acquired dermal melanocytosis OR nevus of Ota-like) AND laser

**10. ClinicalTrials.gov**

Searched on May 27, 2026. No relevant records.

(Hori nevus OR ABNOM OR acquired dermal melanocytosis OR nevus of Ota-like) AND laser

**11. ChiCTR (Chinese Clinical Trial Registry)**

Searched on May 27, 2026. No relevant records.

褐青色痣 OR Hori痣 OR 获得性太田痣样斑 OR ABNOM

*Scopus was not searched due to institutional access constraints; Embase and Web of Science collectively index a comparable scope. Google Scholar search was precluded by regional access restrictions; the four core databases collectively yielded 672 records, making this omission unlikely to introduce meaningful bias.*
