# Supplementary material for: Efficacy of Q-Switched and picosecond lasers for acquired bilateral nevus of Ota-like macules: a systematic review and exploratory dose-response meta-analysis
Source: Lasers Med Sci. 2026 Aug 6;41(1):180. doi: 10.1007/s10103-026-04982-6 (PMC13447452; doi:10.1007/s10103-026-04982-6)
Supplement: Supplementary file 7 [file 10103_2026_4982_MOESM8_ESM.docx]

**Supplementary Table S2. Model-predicted population-average ≥75% clearance rates by treatment session.** *Derived from the dose-response meta-regression (sessions + threshold covariate; cluster-robust SE).*

| **Session** | **Predicted ≥75% Clearance (95% CI)** |
| --- | --- |
| 1 | 4% (2–10) |
| 2 | 13% (6–26) |
| 3 | 32% (16–53) |
| 4 | 60% (37–79) |
| 5 | 82% (65–92) |
| 6 | 94% (85–97) |
| 7 | 98% (95–99) |

*Note: These predictions are derived from an exploratory ecological dose-response model (18 arms, 75 observations). They are purely hypothesis-generating and should NOT be used for clinical decision-making. CIs are model-based and likely anticonservative at upper sessions due to data sparsity.*
