# Supplementary material for: Efficacy of Q-Switched and picosecond lasers for acquired bilateral nevus of Ota-like macules: a systematic review and exploratory dose-response meta-analysis
Source: Lasers Med Sci. 2026 Aug 6;41(1):180. doi: 10.1007/s10103-026-04982-6 (PMC13447452; doi:10.1007/s10103-026-04982-6)
Supplement: Supplementary file 8 [file 10103_2026_4982_MOESM9_ESM.docx]

**Supplementary Table S5. Study-level characteristics of included studies.**

| **Seq** | **Author** | **Year** | **Journal** | **Design** | **N** | **Laser**  **type** | **Wavelength**  **(nm)** | **Device** | **Pulse**  **duration** | **Spot**  **(mm)** | **Fluence**  **(J/cm²)** | **Sessions** | **Interval**  **(wk)** | **Country/**  **Region** | **Lang** |
| --- | --- | --- | --- | --- | --- | --- | --- | --- | --- | --- | --- | --- | --- | --- | --- |
| seq6 | Han R | 2024 | Dermatol Surg | Retrospective | 72 | PS | 730 | Candela Medical (PicoWay) | 246 ps | 2/3 | 2.75-4/1.6-1.8 | 1-3 (mean 2.42) | 27.4 | China | EN |
| seq7 | Zhao W | 2025 | Lasers Surg Med | Retrospective | 49 | PSAL | 755 | Cynosure PicoSure | 750 ps | 2.5-3.3 | 2.34-4.07 | 1-3 (mean 1.7) | 28 | China | EN |
| seq11 | Xie HY | 2024 | Anhui Med Pharm J | RCT | 180 | PS 755 vs QS 1064 | 755/1064 | PS: PicoSure; QS: MedLite C6 | NR | 2-6/3-4 | 0.70-6.37/3.0-9.0 | 5 | 10 | China | CN |
| seq12 | Huang ZY | 2024 | Master's Thesis | Retrospective | 890 | QS | 694/755/1064 | NR | NR | NR | NR | 1-3 | 24-48 | China | CN |
| seq13 | Chen W | 2024 | J Cosmet Dermatol | RCT Split-face | 15 | PS (730 vs 1064) | 730/1064 | Syneron-Candela PicoWay | 250ps/450ps | 2/3 | 2.25-4/2.2-3.7 | 1 | NR | China | EN |
| seq15 | Yang X | 2023 | Skin Res Technol | Retrospective | 110 | QS | 1064 | MedLite C6 (Cynosure) | NR | 3-6 | 2.8-8.0 | 2-9 | 12-24 | China | EN |
| seq20 | Huang L | 2021 | Lasers Med Sci | Retrospective | 482 | QSAL | 755 | Cynosure Accolade | 50 ns | 3 | 5-8 | 2-4 | 6-120 | China | EN |
| seq22 | Phothong W | 2022 | Dermatol Surg | Prospective | 5 | PS vs NS | 1064 | NR | NR | NR | 3.3-3.8/7.2-8.2 | 5 | NR | Thailand | EN |
| seq23 | Zeng JJ | 2021 | Master's Thesis | Retrospective | 126 | QS | 532/1064 | NR | NR | 3-4 | 0.6-1.6/3-5 | 1-6 | 12-24 | China | CN |
| seq27 | Ding H | 2020 | Lasers Surg Med | Retrospective | 225 | PSAL | 755 | Cynosure PicoSure | 750 ps | 2.0-4.5 | 1.26-6.37 | 1-4 | 12-24 | China | EN |
| seq30 | Zeng R | 2019 | Int J Dermatol Venereol | Retrospective | 685 | QS | 1064/755/694 | MedLite C6 / Accolade / SINON | NR | NR | NR | 4.24/2.42/2.65 | ~22 | China | EN |
| seq34 | Hu JP | 2019 | Chin J Aesthetic Med | RCT | 60 | QS Nd:YAG | 532/1064 | NR | NR | 1.5-3/3.5-4.5 | 1.6-2.1/3.0-4.0 | NR | 12 | China | CN |
| seq35 | Su YF | 2018 | Chin J Aesthetic Med | Retrospective | 94 | QS | 1064 | MedLite C6 | 5-10 ns | 3-4 | 3.0-5.0 | 2-7 | 24 | China | CN |
| seq37 | Su YM | 2018 | Master's Thesis | Retrospective | 102 | PSAL vs QSAL | 755/755 | PSAL: PicoSure; QSAL: Accolade | 550-750ps/70ns | 2-4/3 | 1.59-6.37/6.4-7.4 | 2.14/3.00 (mean) | 20-21 | China | CN |
| seq38 | Ma JW | 2018 | Chin J Laser Med Surg | Retrospective | 114 | QSAL | 755 | NR | 60 ns | 3 | 5.5-17.6 | 2-4 | 24 | China | CN |
| seq40 | Wu ZB | 2018 | Chin J Aesthetic Med | RCT | 80 | PSAL vs QSAL | 755/755 | PSAL: PicoSure; QSAL: NR | NR | 2-10/3-5 | 0.20-6.37/3.0-10.0 | 4 | 12 | China | CN |
| seq43 | Yu WY | 2018 | J Am Acad Dermatol | RCT Split-face | 30 | PSAL vs QSAL | 755/755 | PSAL: PicoSure; QSAL: Accolade | 750ps/70ns | 2-2.5/3 | 4.07-6.37/6.0-8.0 | 3 | 24 | China | EN |
| seq45 | Fang HJ | 2017 | Chin Med Cosmetol | RCT | 66 | QS | 1064 | Fotona Q8 | 6-12 ns | 2-3/8 | NR (6-8/2-4 J) | 4.38±2.46/8.53±2.37 (mean) | 12-24 | China | CN |
| seq46 | Huang CW | 2017 | Chin J Aesthetic Med | RCT | 120 | QS Nd:YAG | 532/1064 | Versapulse C (Lumenis) | NR | 3-4/2-3 | 4-5.5/1.8-2.1 | 3+ | 12-24 | China | CN |
| seq47 | Shen JC | 2017 | Chin J Aesthetic Med | Retrospective | 58 | QS | 1064 | STARTR (Korea) | NR | 4 | 5.0-7.8 | 3-5 | 12 | China | CN |
| seq49 | Zhang YJ | 2017 | Hainan Med J | Retrospective | 74 | QS Nd:YAG | 532/1064 | Versapulse C (Lumenis) | NR | 3 | 1.8-2.2/4.5-6.0 | 2-6 | 12-24 | China | CN |
| seq62 | Liu HJ | 2015 | J Hunan Normal Univ Med Sci | Retrospective | 45 | QS Nd:YAG | 532/1064 | NR | 8-10 ns | 1.5-3/3.5-4.5 | 1.6-2.1/3-4 | 1-5 | 12 | China | CN |
| seq66 | Wang Q | 2014 | Chin Prac Med | Retrospective | 33 | QS | 1064 | MedLite C3 (ConBio) | 10 ns | 2-4 | 3.5-5 | 1-5 | 12-24 | China | CN |
| seq76 | Ge HM | 2014 | Chin J Aesthetic Med | Retrospective | 20 | QS (Low energy) | 1064 | Lutronic (Korea) | NR | 6 | 2.2-3.0 | 10 | 3 | China | CN |
| seq79 | Zhang T | 2013 | Chin J Aesthetic Med | RCT | 52 | QS Nd:YAG | 1064 | Jilin Koying | 6-12 ns | 8/2-3 | NR (400-1400 mJ) | 8.68/4.77 (mean) | 12-24 | China | CN |
| seq82 | Meng J | 2012 | Chin Med Cosmetol | Retrospective | 56 | QS | 1064 | MedLite C6 | 5-20 ns | 3/4 | 6.0-8.5 | 3-5 | 12-24 | China | CN |
| seq97 | Qu XY | 2011 | Chin J Lepr Skin Dis | Retrospective | 103 | QS | 1064 | MedLite II (Conbio) | 5-10 ns | 2-3 | 3.5-12.0 | 1-4 | 12-24 | China | CN |
| seq98 | Hong X | 2011 | Chin J Aesthetic Med | Retrospective | 270 | QS | 1064 | Jilin Yingke | NR | NR | 4.5-8 | 1-3 | 4-12 | China | CN |
| seq103 | Ding XD | 2010 | J Commun Med | Retrospective | 86 | QS | 1064 | Chongqing Jingyu | 8 ns | 2-3 | 0.65-0.9 | 4-8 | 12-24 | China | CN |
| seq104 | Mu YZ | 2010 | Chin J Aesthetic Med | Retrospective | 165 | QS Nd:YAG | 532/1064 | Versapulse C (Lumenis) | NR | 3 | 1.8-2.2/4.5-6.0 | 2-5 | 12-24 | China | CN |
| seq105 | Huang WP | 2010 | Chin J Integr Med | Retrospective | 72 | QS | 1064 | MedLite C3 (ConBio) | 10 ns | 2-4 | 3.0-4.0 | 2-5 | 12-24 | China | CN |
| seq120 | Yuan LS | 2007 | Chin J Aesthetic Med | Retrospective | 180 | QS Nd:YAG | 1064/532 | NR | 10 ns | 1-3 | 2-6 | 2-4 | 12-24 | China | CN |
| seq126 | Yin F | 2006 | Chin J Pract Aesth Plast Surg | Retrospective | 38 | QSAL | 752 | Cynosure | 45-75 ns | 2.4 | 6.5-9.0 | 3-5 | 8-12 | China | CN |
| seq136 | Li CH | 2004 | Chin J Laser Med Surg | Retrospective | 202 | QSAL | 755 | Versapulse (Lumenis) | 100 ns | 3 | 6.0 | 1-5 | 12 | China | CN |
| seq137 | Wang HW | 2004 | J Clin Dermatol | Retrospective | 43 | QSAL | 755 | NR | NR | 3 | 7.2-8.8 | 1-4 | 8-12 | China | CN |
| seq144 | Lam AY | 2001 | Dermatol Surg | Retrospective | 32 | QSAL | 755 | NR | 100 ns | 3 | 8 | 2-13 (mean 7) | 4.7 (mean) | Hong Kong | EN |
| seq146 | Kunachak S | 2000 | Lasers Surg Med | Prospective | 70 | QS | 1064 | Lambda Photometrics | 25 ns | 2/4 | 8-10 | 2-5 (mean 2.8) | 2-3 | Thailand | EN |
| seq147 | Polnikorn N | 2000 | Dermatol Surg | Retrospective | 66 | QS Nd:YAG | 1064 and 532 | NR | 6-8 ns | 3 | 4-6 | 1-7 | 12-24 | Thailand | EN |
| seq152 | Cao N | 2019 | Chin Med Cosmetol | RCT | 104 | QSAL 755 vs QS 1064 | 755/1064 | QSAL: Cynosure; QS: STARTR | 50 ns/NR | 3/4 | 7.2-8.0/5.0-7.8 | NR | 24 | China | CN |
| seq160 | Evangelista KER | 2021 | Dermatol Surg | Prospective | 22 | PS | 670 | Cutera Enlighten | NR | 4 | 0.9 | 1 | NR | Thailand | EN |

*NR, not reported; RCT, randomized controlled trial; PS, picosecond; QS, Q-switched; AL, alexandrite; Nd:YAG, neodymium-doped yttrium aluminum garnet. "PS vs QS" indicates parallel-group or split-face designs. Device names as reported in original publications.*
